# Mechanical and Magnetic Properties Variation in Non-Oriented Electrical Steels with Different Cutting Technology: A Review

**DOI:** 10.3390/ma17061345

**Published:** 2024-03-14

**Authors:** Gheorghe Paltanea, Veronica Manescu (Paltanea), Aurora Antoniac, Iosif Vasile Nemoianu, Horia Gavrila

**Affiliations:** 1 Faculty of Electrical Engineering, National University of Science and Technology Politehnica Bucharest, 313 Splaiul Independentei, District 6, 060042 Bucharest, Romania; 2 Faculty of Material Science and Engineering, National University of Science and Technology Politehnica Bucharest, 313 Splaiul Independentei, District 6, 060042 Bucharest, Romania; antoniac.aurora@gmail.com; 3 Technical Sciences Academy of Romania, 26 Bulevardul Dacia, 030167 Bucharest, Romania

**Keywords:** cutting technologies, electrical steel, hardness, crystallographic structure, magnetic properties

## Abstract

The problem of energy consumption reduction establishes important challenges for electric motor producers in the framework of new international regulations regarding the conditions that must be accomplished by motors in the near future. One of the most important topics is related to the core loss decrease directly linked to the effect of electrical steel degradation induced by the cutting technology. Understanding exactly how this phenomenon occurs by analyzing the chemical, mechanical, crystallographic, magnetic domain, and magnetic properties is of utmost importance when manufacturing processes must be changed and adapted to a new market characterized by high-efficiency motors. Today, mechanical and laser cutting technologies are the most used because of their reduced price and high-speed process. Still, unfortunately, these methods are not the best due to the fact that they lead, in most cases, to a high value of magnetic core losses, low electromagnetic torque, and hence reduced efficiency. This review paper shows that non-conventional technologies such as water jetting and electroerosion could be applied if proper modifications are added. This paper’s main idea is to present a comprehensive study regarding the impact of cutting technologies on microhardness and residual stresses, crystallographic texture, magnetic domain structure, and magnetic properties of some non-oriented electrical steels used in motor production. It provides a detailed analysis of the abovementioned aspects by including the authors’ research and findings in the wider context of other research group contributions. It also offers a general idea of the mechanisms present at the macro- and microscopic levels. The readers can find some of the most used analytical models, including the cutting process’s damaged effect on the magnetic properties’ variation based on a simple mathematical approach and examples of finite element modeling performed on real motor designs implemented in various programs. Last but not least, some practical implementations of the cutting procedure’s influence on motor working conditions are presented in the last section of the paper. It provides an up-to-date analysis regarding how the cutting method should be included in high-efficiency motor production by emphasizing the importance of the topic and identifying where supplementary research must be undertaken. From the investigated literature, by analyzing specific sample geometries associated with different characterization methods, it can be concluded that all the cutting technologies have an important contribution to the mechanical and magnetic quantities. When the magnetic core of an electric motor is produced through non-conventional methods, the overall influence of the cutting procedure has a low percentage in the motor efficiency, as presented in this paper.

## 1. Introduction

Nowadays, the problem of climate change has forced a lot of electrical motor producers to respect a list of energy requirements found in the European Union (EU) under the name of Ecodesign [1,2]. Starting in July 2023, the third step of this process begins, stating that motors with a rated power between 0.75 kW and 1 MW must comply with IE3 (premium efficiency) class, except the ones in the range of 75 kW to 200 kW that must meet the IE4 (super premium efficiency) class, while motors characterized by an electric power between 0.12 kW and 0.75 kW have to respect the regulations of IE2 (high efficiency) class. Eight billion electric motors were counted inside the EU, and more than half of worldwide energy consumption occurs only in Europe. It is expected that if the regulations of Ecodesign are respected, an annual saving sum of about 110 TW will occur by 2030. In addition, 40 million tons of CO_2_ per year will be avoided [1]. Outside the EU exists a plan called Super-Efficient Equipment and Appliance Deployment (SEAD), which promotes using energy-saving motors and other devices. The idea of a world full of highly efficient motors and increased awareness regarding climate influences is sustained today by the International Energy Agency’s 4E Electric Motors Systems Annex, which provides the mechanisms that must be followed during the process of high-efficiency motor introduction. Dems et al. [3] showed that reducing motor loss is essential if the new regulations regarding energy consumption are considered. They noticed that in the case of motors working at low and industrial frequencies, although the core losses are relatively high, the most important part of the total energy losses is due to the motor windings. On the other hand, for high-frequency range motors, core losses are prevalent. De Almeida et al. [4] proved that the core losses increase was directly proportional to the variation in motor power. Supplementarily, the stray load loss percentage was higher, and the authors concluded that this parameter depends on different factors, such as classical core losses.

It can be foreseen that decreasing the core losses in electrical motors is a problem of high priority worldwide, and it can be considered and assessed through different strategies by the motor producers. One strategy discussed in this review paper is based on the fact that the practical implementation of an electric motor depends on the cutting procedures that are applied to obtain a certain geometry of the motor’s magnetic core. The influence of cutting technologies on the macroscopic properties such as microstructure, mechanical characteristics, and magnetic properties of non-oriented electrical steels is well known, and it has been extensively investigated in the literature [5,6,7,8,9]. It was found that an important deterioration of the crystallographic structure and an increase in the energy losses occurs when the cutting perimeter increases, as well as a modification of the material magnetization process, which generates the necessity of higher magnetic field strength (*H*) application to obtain the same value of magnetic flux density (*B*) [10,11]. Regarding the non-oriented electrical steels (NO FeSi), the total energy losses and magnetic flux density are important characteristics that are discussed in the literature [12], having an influence on the motor efficiency and torque [13]. In addition, grain sizes, defects, inclusions, precipitates, and grain orientations are important features in the control of magnetic properties of NO FeSi, being considered variable as a function of steel chemical composition [14,15], cutting technology [16], thermomechanical processing [17,18], and coating material [19].

The most used technologies involved in ferromagnetic lamination fabrication are mechanical (punching or guillotine), laser, waterjet, and electroerosion cutting. Punching represents a common method for motor manufacture at the industrial level in mass production due to its reduced cost and increased speed [20,21]. Its main drawbacks are the bluntness of the cutting tool and burr presence at the cut edge, which is directly linked to a lower stacking factor. Many studies [22,23,24,25] investigated the invasive character of laser cutting technology in comparison to mechanical punching regarding the deterioration of material properties. An overall agreement established that edge rounding and stress hardening phenomenon at the cutting edge occur in the case of punched strips. In contrast, thermal stresses are present on the laser-cut edge. Both cutting technologies induce an important deterioration of macroscopic properties of electrical steel in comparison to less damaging methods such as waterjet (WJ) [26] and electrical discharge machining (EDM) [27,28]. Winter et al. [29] analyzed the effect of pulsed laser, abrasive waterjet, wire electrical discharge machining, and continuous wave laser methods on the magnetic properties of non-oriented 2.4 wt.% Si ring samples with 0.2 mm thickness. The main conclusion was that the abrasive waterjet machining determined the lowest core losses and applied magnetic field strength since the continuous wave laser induced the highest core losses and lowest saturation magnetic flux density. Emura et al. [22] investigated four cutting technologies, including guillotine, photo corrosion, laser, and punching, used to cut a 2 wt.% Si NO FeSi with a thickness of 0.5 mm. The highest magnetic permeability value and the lowest losses were measured in the case of photo corrosion cut samples, and the laser-cut materials exhibited the highest coercive force and the lowest remanent magnetic flux density. The previously presented analyses [22,27,28,29], evidenced that the wire cut method is characterized by a minimum impact on the magnetic properties of the electrical steel but concomitantly showed its main drawbacks such as sample burned coating due to local high-temperature apparition [30] and low cutting speed [31]. Paltanea et al. [32] compared mechanical and waterjet cutting techniques’ effects on NO FeSi with 0.5 mm and 0.65 mm thicknesses. They analyzed the magnetic properties such as total energy losses, saturation induction, magnetic permeability, remanent magnetic polarization, and coercive field of the electrical steel at the industrial frequency of 50 Hz. They found that the waterjet cutting technique led to the most reduced energy losses and coercive field and the highest magnetic permeability and remanent polarization values. Table 1 summarizes some of the main advantages and drawbacks of the cutting technologies mentioned above [33,34].

**Figure 1:**
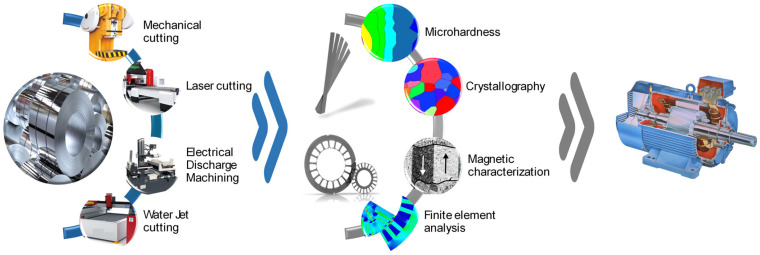
Process diagram showing the main electrical steel cutting technologies and pointing out the laboratory investigations that are useful to analyze the cutting procedure influence on material properties to design high-efficiency motors.

This review paper investigates the cutting influence of different technologies on the motor’s core features, considering the material property deterioration and models that describe this phenomenon. The chemical composition of some industrial grades of electrical steel, residual stresses generated by the cutting procedure concomitantly associated with the microhardness increase, the magnetic domains, crystallographic structure modifications, and grain sizes and shape variations will be analyzed. Further, the authors will focus their attention on magnetic properties by describing experimental results in quasi-static and dynamic conditions and analytical and numerical models used to estimate the magnetic properties changes and magnetic losses near the cut edge. Last but not least, there will be provided some examples of electric motors with magnetic cores prepared based on different cutting technologies by discussing them in detail. the authors’ results and other researchers’ findings will be included in the paper’s sections to sustain the investigated topic. Figure 1 presents a graphical chart starting with the electrical steel cutting technology and then showing the cutting procedure effects analysis necessary to design high-efficiency electrical motors.

## 2. Main Grade and Chemical Composition of Non-Oriented Silicon Iron Strips

In this section, the main chemical elements’ roles will be discussed together with examples extracted from the literature with chemical compositions determined by various research groups [35,36,37,38,39,40,41,42,43]. Non-oriented electrical steels exhibit an almost isotropic grain texture and are included in the soft magnetic material class, mainly used for electric machine core manufacturing. Usually, they are sold in a large variety of commercial grades, classified as a function of silicon (Si) content and losses. NO FeSi properties and specifications are given in two international standards (IEC 60404-8-2 [44] and IEC 60404-8-4 [45]). As a general observation, the Si content comprises between 1 and 3.7 wt.%. The other chemical elements included in the alloy structure are aluminum (Al) (0.2–0.8 wt.%) and manganese (Mn) (0.1–0.3 wt.%). These two elements have an important contribution to the increase in mechanical properties and alloy resistivity. Aluminum is used to decrease the aging process generated by nitrogen (N) precipitation phenomenon through AlN second phase apparition [46].

Higher grades of NO FeSi are sold by the producers as fully processed materials. Firstly, hot rolled sheets with an approximative thickness of 2 mm undergo a cold rolling process; then, they are annealed at an average temperature of 800 °C and reduced to the final thickness with a minimum value of about 0.35 mm. After that, a decarburization and recrystallization annealing at 750–900 °C is performed, followed by a final grain-growth annealing at 850–1100 °C. These materials are coated with chromate- or phosphate-based layers, providing a good lamination-cutting possibility. The lower NO FeSi grades contain a maximum Si percent of about 2 wt.% being manufactured and delivered as semi-processed products. During the production stages, one can notice firstly a melting, degassing, and casting of the sheets, followed by a re-heating at 1000–1250 °C and a hot rolling procedure when they achieve a thickness of about 0.65 and 0.50 mm. No stress relief treatments are applied for low-quality grades [46].

Through analyzing and controlling parameters such as crystallographic texture, grain sizes, impurities, residual mechanical tensions, and surface quality one can improve the NO FeSi quality. It was noticed that only a small percent of a few ppm of carbon (C), nitrogen (N), sulfur (S), and oxygen (O) could lead to deterioration of magnetic properties, such as increased values of the coercive field and energy losses. This fact is due to precipitate apparitions acting as pinning sites for magnetic domain movements. In addition, the precipitates can have an unwanted effect on the grain growth process [46]. During the fabrication process, the impurity percent must be kept within certain limits to permit grain growth with an optimal size between 100 and 200 μm. It was found that MnS and AlN have an important influence on the formation of a detrimental crystallographic texture characterized by {111} plane existence [35]. Antimony (Sb) and tin (Sn) are impurities that determine the re-crystallized grain growth to an almost ideal cubic texture {100}<0vw>. An increased Al content is associated with subsurface oxidation, which appears during the decarburization step. For these types of alloys, it is mandatory to perform the decarburization and denitrogenization of the melt in vacuum conditions [35].

Table 2 presents some NO FeSi grades with different chemical compositions extracted from the literature.

The chemical composition is very important because it has a definitive contribution to electrical steels’ mechanical and magnetic properties. It is advised to continuously search for new chemical element additions to improve the alloy quality and to provide the electric motor producers with adequate materials for efficient motors.

## 3. Morphology of Local Deformation Generated by Cutting Technology at the Cut Edge and Macroscopic Properties Modification

In this section, the influence of cutting technology on local changes in the material microhardness and residual stresses, crystallographic texture and microstructural features, and magnetic domain structure will be emphasized. As mentioned above, it can be noticed that the methods that have a significant impact are punching, shearing, and laser cutting, which are used in the large production of electrical motors. Regarding the less invasive technologies (WJ, EDM), a reduced deterioration of material structure and properties can be experimentally observed being influenced by different conditions. Considering the high costs and special resources involved, these methods are used only for prototyping or small series production.

Spadlo et al. [47] investigated the effect of cutting speed for the WJ method on local temperature changes for the structural steel microstructure. They have chosen three different cutting speeds (250 mm/min, 300 mm/min, 350 mm/min) and water jet pressures (380 MPa, 340 MPa, 300 MPa), while the specimens were cut at a constant value of abrasive flow rate (250 g/min). The authors observed that during the cutting process, the material temperature increased due to important changes in internal energy generated by plastic deformation. They concluded that the maximum value of the indentation microhardness measured at 14 μm from the cut edge was about 3750 MPa obtained for 250 mm/min and 380 MPa since the minimum value (3300 MPa) was achieved for 300 mm/min and 340 MPa. High plastic deformation was evidenced at high pressure and low cutting speed, being directly linked with a temperature increase at about 450 °C. In addition, Bankowski et al. [48] showed that an increased temperature appeared on the cut edge due to the above mentioned phenomenon. The authors set the pump pressure at 380 MPa and the cutting speed at 50 mm/min. The nanohardness measurements evidenced that the hardness value in the jet impact zone was about 620 HV compared to the base metal (423 HV) sustained by an important heat transfer and plastic deformation at the cut edge. One can conclude that the cutting speed is an important parameter in the WJ method.

Krahmer et al. [49] investigated alternative processes to mechanical cutting, such as laser, WJ, and EDM, in the case of low-carbon steel plates. They found that WJ technology induced a tapper error, which led to a non-uniform sample thickness. However, this method did not influence the edge ductility and the mechanical properties, being a suitable and medium-cost alternative to mechanical cutting. Some weak points, such as a very slow process and initial preparation requirements, were indicated regarding the EDM technology. Its major advantage was that the cutting edge met the geometrical conditions of the sample with no changes in mechanical properties and material ductility. The authors stated that laser technology should be used only if necessary because of the increased hardness at the cut edge.

### 3.1. Microhardness and Residual Stresses

The microhardness test consists of a small indenter that is applied under different loads’ action on a material surface [50]. This method is easy and fast and permits an estimation of the strain-hardening effect, which occurs at the cut edge zone. Many research publications analyze the impact of cutting technology on the material microhardness [36,37,38,41,51]. Some of the most important findings are summarized.

**Figure 2:**
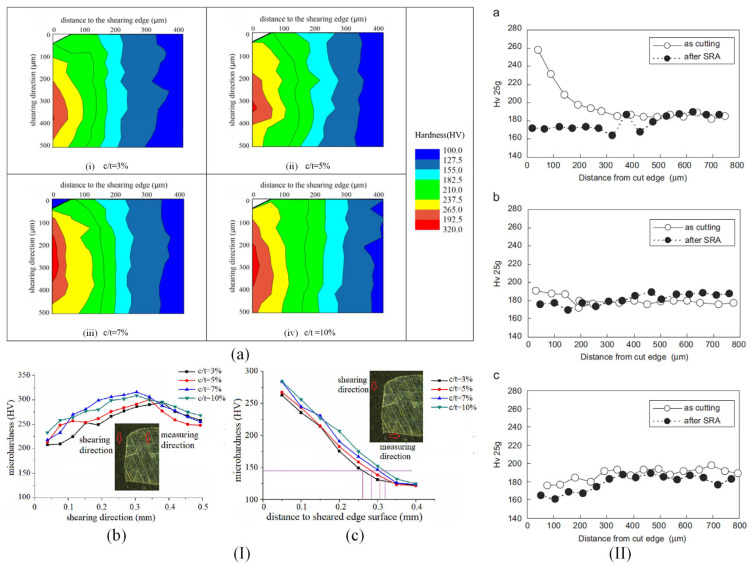
Vickers microhardness experimental results. (**I**) MS101 electrical steel (2.9 wt.% Si, thickness 0.5 mm) cut through shearing with different clearance distances: (**a**) microhardness nephogram, (**b**) hardness values measured along the shearing direction, and (**c**) perpendicular to the shearing direction [38]. Figure is licensed under CC—by 4.0. (**II**) Hardness values and profiles measured near the cut edge for different cutting technologies: (**a**) shearing, (**b**) laser cutting, (**c**) wire electrical discharge machining [54]. Reprinted from [54] Copyright (2024), with permission from Elsevier.

Omura et al. [51] investigated the influence of shearing technology on samples prepared from non-oriented electrical steels with a thickness between 0.20 and 0.50 mm, a hardness of HV150-220, and an average grain size of 70 μm. The effect of hardness was evaluated based on a maximum of 5 samples, cut with a variable width of 5 and 30 mm, parallel or perpendicular to the rolling direction. Small pieces were cut and inserted in resin, and then Vickers microhardness was measured at intervals of 40 or 50 μm inside the sheared edge. A load of 50 g was applied, and a holding time of 10 s was set. Measurements were repeated five times by choosing the perpendicular direction to the cut edge. It was observed that the hardness of the material increased near the cut edge, having a maximum value of about 60% (HV0.5):154 and 50% (HV0.5):217 and then gradually decreasing to about 0% for a distance from the sheared edge of about 0.5 mm. This high hardness value was due to the plastic deformation that occurred near the cut edge. It was estimated that the plastic deformation region is about half of the material thickness, and it was concluded that the shearing process induced elastic stresses in a zone with a width three times the sample thickness. Saleem et al. [36] analyzed 35WW300 non-oriented electrical steel cut based on laser technology. The samples were polished successively using 600, 800, and 1200 SiC grit papers, followed by a cloth polishing based on 3 μm and 1 μm oil-based diamond suspension and a vibratory polishing involving 0.05 μm colloidal silica suspension for 20 h. The nanoindentation measurements were made with a calibrated Berkowich diamond indenter tip by applying a maximum force of about 5000 μN with a hold time of 2 s. The indentation process was performed along a row starting near the cut edge with a space of 30 μm between two indents. The authors suggested that the laser cutting procedure did not induce a mechanical hardening effect near the cut edge, finding a constant value for hardness of about 3.5 GPa. Wang et al. [37] measured the hardness of NO FeSi with a thickness of 0.30 mm, cut through punching with a grain size of 27 μm, by varying the instrument clearance between 10 μm and 35 μm, with a step of 5 μm. The sample microhardness was evaluated based on the Vickers method. Near the cut edge, a maximum value of 430 HV_0.1_ at 35 μm clearance and a minimum value of 370 HV_0.1_ at 25 μm clearance were found. The authors estimated the hardness of the original material at 243 HV_0.1_. It was concluded that initially, the cutting process led to an increase in microhardness value at the cut edge, followed by a gradual decrease until the original material hardness was attained. The main finding of the paper was that a clearance of 25 μm was linked to the lowest value of the hardness being correlated with a much more reduced plastic deformation effect. Weiss et al. [6] investigated non-oriented electrical steels with Si content of 2.4 wt.% and thickness of 0.35 cut through the shearing process. The virgin material Vickers hardness was estimated at about 171 HV_0.2_. The authors modified the cutting speed (0.04 and 0.15 m/s) by keeping the clearance constant at 30 μm. For the experimental measurements, they applied a Vickers spike with a force of 0.25 N. A maximum value of mean normalized hardness of about 1.7 in the case of a worn cutting tool at 0.15 m/s measured near the cut edge, at 90° to the rolling direction, was found. The minimum value of 1.52 was noticed for a sharp cutting tool at 0.04 m/s determined in the same conditions. In addition, it was noticed that at a distance higher than material thickness, the mean normalized hardness values became equal to 1. Zhu et al. [38] analyzed the effects of shearing processing on electrical steel MS101 with a thickness of 0.50 mm. The tool lateral clearance was in the 0.01–0.060 mm range, with a relative lateral clearance (c/t) between 2% and 12%, and a shearing speed of 0.28 m/s. The shearing edge radius was equal to 23 μm. The measurements performed along the shearing direction evidenced a maximum microhardness value of about 300 HV for a relative lateral clearance of 7% and a shearing direction of 0.3 mm. In the case of c/t = 3%, a maximum value of about 275 HV was obtained at a shearing direction of 0.38 mm. Taking into consideration the measurements made as a function of the distance to the sheared edge surface, a maximum value of 285 HV can be noticed for c/t = 10% and 7% since a minimum value of 265 HV was measured in the case of c/t = 3% and 5% (Figure 2I). The authors of this study concluded that the relative lateral clearance is an important parameter that must be considered when detailed analyses are necessary.

Hofmann et al. [41] analyzed M300-35A NO FeSi industrial grade samples cut with guillotine, laser, and spark erosion technologies. The local microhardness measurements were made with a 50 g μ-Vickers indenter localized at the cut edge starting at 25 μm. The measurements were spaced at intervals of 100 μm at the sample surface placed on parallel lines. A minimum lateral resolution of 10 μm was achieved at the cut edge. It was estimated that the bulk material has a value of about 235 HV. The results obtained near the cut edge were as follows: 235 HV—laser cut, 360 HV—guillotine, and 210 HV—spark erosion. It can be noticed that the laser technology did not produce a mechanical hardening effect on the sample surface, while the guillotine cutting performed with a clearance of 0.02 mm generated an increased hardness of about 360 HV associated with a hardened zone of 150 μm. On the contrary, the spark erosion induced a softening effect of the cut edge being related to a hardness lower than that of the bulk material of about 210 HV and a width of 120 μm. Shi et al. [52] prepared samples of high-grade non-oriented electrical steel sheets (35W270) containing 3 wt.% Si using punching, shearing, laser cutting, wire-EDM, and abrasive WJ technologies. They set the following cutting conditions: 0.1 mm clearance for punching, 0.05 mm clearance for shearing, CO_2_ laser with a power of 1500 W, pulse frequency of 100 Hz, cutting speed of 8 mm/min—for laser, 0.2 mm brass wire and cutting speed of 30 mm^2^/min for wire-EDM, and water press of 300 MPa and a cutting speed of 2 m/min for abrasive WJ. A stress relief annealing treatment (SRA) was applied after the cutting process at 750 °C for 2 h in N_2_ atmosphere. Vickers hardness determinations evidenced the presence of plastic deformation structure and burr apparition in the case of punching linked with a hardness value of about 460 HV (load 10 g, no SRA applied) and 260 HV (load 10 g, SRA applied). For shearing technology, the authors obtained a value of 360 HV and 200 HV in the same conditions. The abrasive WJ cutting and wire-EDM had similar results for hardness determined near the cut edge of about 260 HV before the SRA and about 220 HV after the SRA. The laser cutting method led to the lowest hardness values, obtaining about 240 HV before SRA and about 220 HV after SRA. At a distance of 2400 μm, the hardness values for all the samples were equal to 270 HV in the absence of SRA. It was concluded that the cutting procedure had an important influence on the sample hardness near the cut edge. Araujo et al. [53] cut NO FeSi using laser and mechanical technologies. The Vickers microhardness measurements were triplicated along two lines perpendicular to the cut edge. A maximum value of 225 HV was obtained for the mechanical cut samples near the cut edge, while a constant value of 175 HV was measured for the laser technology. Kurosaki et al. [54] prepared samples from 50A470 JIS non-oriented electrical steel with 2.0 wt.% Si and 0.3 wt.% Al based on shearing (clearance of 5%), laser cutting (CO_2_ laser, 200 W, 5 m/min, pulse frequency 100 Hz), and wire-EDM (0.2 mm diameter brass wire, 30 mm^2^/min). After the cutting process ended, the SRA treatment was applied. The hardness of shearing samples measured near the cut edge was equal to 260 HV 25 g before SRA and 175 HV 25 g after SRA. For the other two technologies, the following results were obtained: laser—190 HV 25 g (before SRA) and 175 HV 25 g (after SRA), wire-EDM—175 HV 25 g (before SRA) and 165 HV 25 g (after SRA) (Figure 2II). The authors noticed that the SRA treatment did not influence the laser or wire-EDM cutting technologies in a high amount. In contrast, in the case of the shearing method, this treatment was considered highly efficient in reducing the strain-hardening phenomena.

The main conclusion, which can be extracted from all the abovementioned studies regarding the cutting technology influence on the NO FeSi steel hardness, consists of the fact that mechanical cutting determines the highest increase in the hardness value near the cut edge, while the laser produces a negligible effect. Regarding the wire-EDM and abrasive-WJ methods, a reduced value of hardness in comparison with the reference characteristic for each material is experimentally measured, so as a direct consequence, the softening effect of these two technologies must be pointed out. The stress relief annealing treatment applied at a temperature of about 750 °C for 2 h in an N_2_ atmosphere reduces a high amount of the plastic deformation effects in the case of mechanical cutting, exhibiting the lowest impact on the other three technologies.

**Figure 3:**
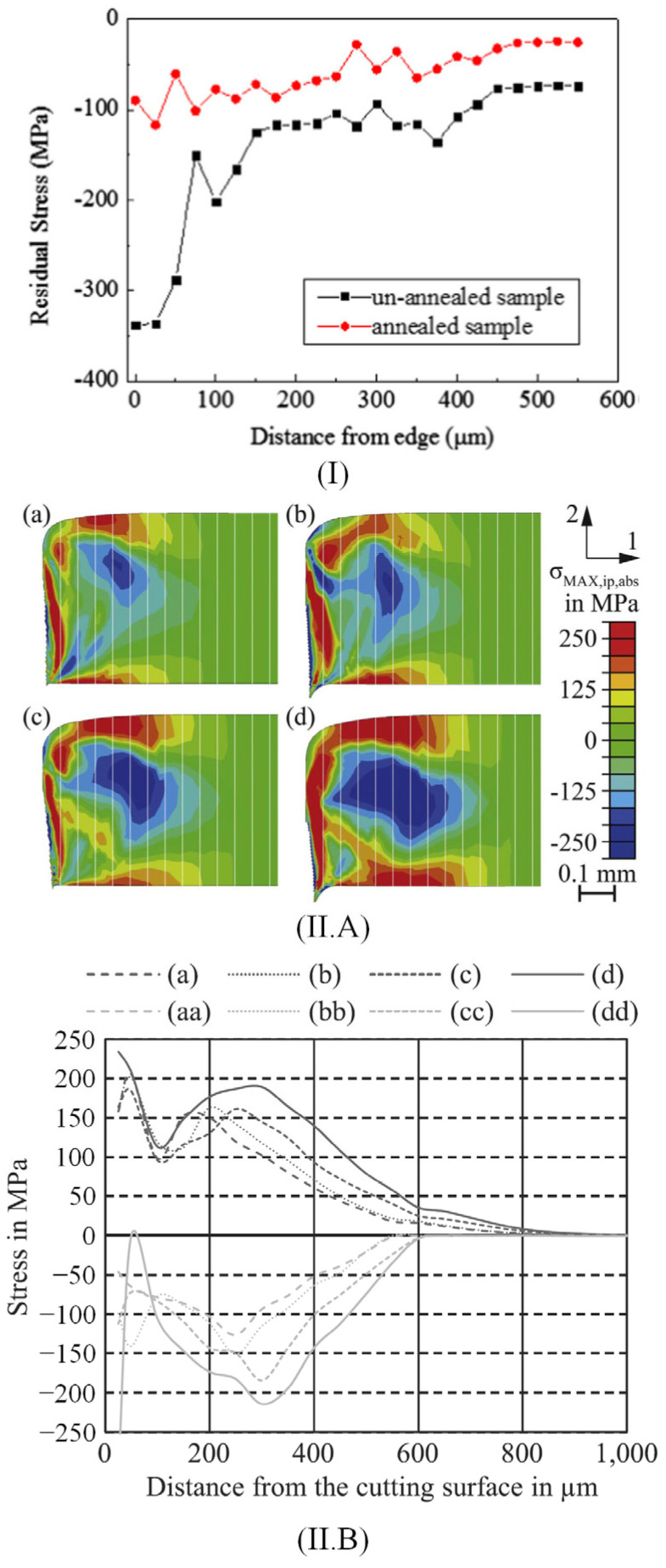
Residual stress experimental and numerical results: (**I**) Comparison between residual stress values measured through the nanoindentation method on as-cut and annealed samples [7]. Reprinted from [7] Copyright (2024), with permission from Elsevier; (**II**) Finite element analysis of mechanical cutting in the case of (**a**) sharp tool with 10 μm clearance, (**b**) worn tool with 10 μm clearance, (**c**) sharp tool with 30 μm clearance, (**d**) worn tool with 30 μm clearance: (**A**) residual stress distribution maps and (**B**) tension (a, b, c, and d) and compression (aa, bb, cc, and dd) residual stress mean values computed along the strip thickness in 90° to rolling direction [6]. Reprinted from [6] Copyright (2024), with permission from Elsevier.

As previously mentioned, plastic and elastic deformations and residual stresses due to the cutting procedure have an important influence on the degradation of the material’s macroscopic properties. This phenomenon is emphasized at low strains below 5% and in the low and medium magnetic field strength domain of kA/m order [55,56,57] (Figure 3). In Table 3 are summarized some studies, which analyze the residual stress distribution and hardness values near the cut edge.

### 3.2. Crystallographic Texture and Material Structure

Crystallographic structure and texture are other important parameters that must be considered when the morphology of the local deformation is analyzed [14,58,59]. The main experimental methods to investigate them are electron backscatter diffraction (EBSD), which provides the alloy texture, and optical microscopy, which offers the possibility to visualize the surface microstructure [60] and to determine through metallographic measurements the average grain size and the grain distribution [61].

**Figure 4:**
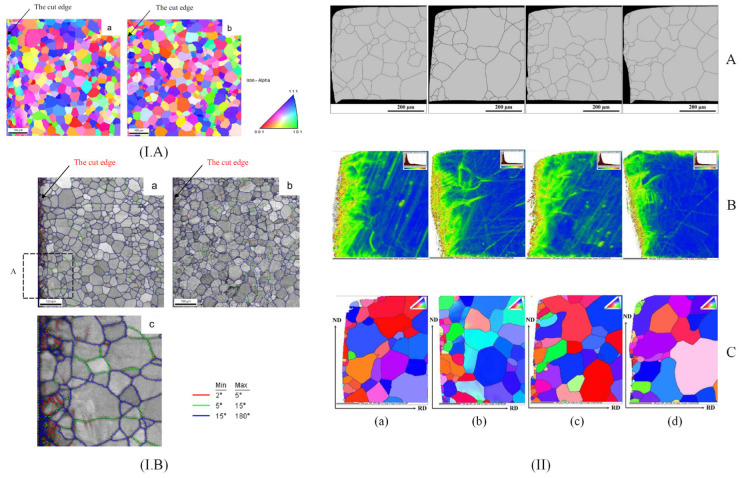
Microstructural optical observations and EBSD investigations of mechanically cut NO FeSi: (**I**) Crystallographic texture near the shear cut edge for samples of 0.5 mm thickness: (**A**) EBSD orientation image maps for (**a**) upper and (**b**) lower surfaces of the strip and (**B**) SEM images in the case of (**a**) upper and (**b**) lower surfaces of the strip, and (**c**) zoom of region A pointing out in colors the range of different angle distributions [62]. Reprinted from [62] Copyright (2024), with permission from Elsevier; (**II**) Crystallographic investigations of the shear cut profile on M350-50A grade steel: (**A**) optical images after SRA treatment, (**B**) local misorientation maps before SRA and (**C**) inverse pole figure maps after SRA in the case of (**a**) 1%, (**b**) 3%, (**c**) 5%, and (**d**) 7% cutting clearance [39]. Figure is licensed under CC—by 4.0.

Xiong et al. [62] studied the texture evolution in non-oriented silicon iron strips mechanically cut. The orientation EBSD maps determined for the cut edge area evidenced that the grains were not uniformly distributed because the plastic deformation generated during the mechanical cutting process was directly linked to the grain crystalline orientation rotation (Figure 4I). In Figure 4(IA) is presented the misorientation angle distribution near the cut edge. It can be concluded that the mechanical cutting generated a significant increase in low angle boundaries (LAGBs) (θ between 2° and 15°) from the upper and lower surfaces, respectively. The misorientation distributions for these regions are different because the upper surface exhibited a higher percent of LAGBs concomitantly with reduced boundary misorientations that are changed as a function of distance measured from the cut edge in a much more noticeable way in comparison with the lower surface. In order to investigate the material texture evolution (Figure 4(IB)) located on the upper surface, the φ_2_ = 45° section analysis was performed. It was concluded that the mechanical cutting led to a clear texture gradient from the cut edge to the material central zone by decreasing the intensity of γ ({111}<uvw>) and λ ({001}<uvw>) fiber components. Füzer et al. [39] investigated the texture evolution and crystallographic structure in NO FeSi strips of industrial grade M350-50A cut through punching with different cutting clearances. The microstructure was analyzed based on optical microscopy and EBSD (Figure 4II) performed on a metallographic cross-section along the rolling direction. A bimodal distribution of ferritic grains, with an average size ranging between 77.2 μm and 87.9 μm was evidenced. The authors concluded that strong mechanical deformations associated with annealing heat treatments led to microstructure refinement in the cut-edge vicinity. The average local misorientation map obtained after specific EBSD analyses showed an increased misorientation grade near the cut edge before SRA. In addition, the cutting clearance influence analysis showed that smaller values can reduce the residual stress depth but can increase the stress values near the cutting surface.

Araujo et al. [53] investigated the crystallographic texture and misorientation gradients of 0.5 mm thick NO FeSi laser and mechanical cut based on EBSD. Their analysis evidenced the texture evolution, which began near the cut edge in the case of a sample cut through laser technology by analyzing the section φ_2_ = 45° of an orientation distribution function (ODF) given by EBSD. The ODFs were computed by including all the grains situated within a distance of 50 μm (A zone) and 140 μm (B zone) as well as in the middle of the sample. It was noticed that inside the A and B areas, the orientations were away from the classical gamma fiber ({111}<uvw>), and the intensity lines located in the ODFs of zone A were concentrated along the {h11}<1/h,1,2> fiber. For the mechanical cut samples, it was difficult to achieve a good quality diffraction pattern indexation with EBSD technology due to the heavy cold deformation. In addition, gradients and local orientation changes were not visible. Shi et al. [52] analyzed the effect of different cutting methods such as mechanical, laser, wire-EDM, and abrasive WJ at φ_2_ = 0° and φ_2_ = 45° sections based on the ODF method before and after SRA treatment. They observed, before the SRA application, that the most important texture components in the non-affected area are placed on {110}<665> and near {310}<130>; water jet cutting produced a simple texture that contained only {110}<112>, while the laser technology generated {113}<172> texture. Regarding the punching procedure, a similar effect of a strong variation in grain orientation due to lattice deformation, also described in [53], was noticed. The authors concluded that before SRA, the texture of mechanically cut samples was the most complex and difficult to analyze. After SRA treatment, it was observed that the texture of all investigated samples became much simpler, and the orientations specific to laser-cut samples observed near the cut edge disappeared. By analyzing the cut edge generated by punching, only {940}<490> and {221}<114> components were noticed.

Considering all the studies described above, it can be concluded that the cutting method has an important influence on the crystallographic texture and structure. Mechanical cutting usually determines an increase in low-angle boundaries, and the tool clearance can reduce the residual stress depth. Many researchers reported the impossibility of obtaining high-quality EBSD maps for shearing and punching. Regarding the laser method, it was noticed that there was an important influence on the grain texture located near the cut edge due to thermal stress and heat-affected zones (HAZs), while the abrasive WJ and wire-EDM technologies led to the most simple grain texture from all the methods.

### 3.3. Magnetic Domain Structure

Magnetic domain structure can be investigated based on magneto-optic microscopy, which usually uses conventional imagining optics combined with the Kerr effect (MOKE). It consists of linearly polarized light rotation in correlation with the magnetization vector direction on reflection from an opaque sample.

Takezawa et al. [63] investigated the effect of strain-induced through mechanical cutting in NO FeSi electrical motor tooth. They used a Kerr microscope to analyze the magnetic domains before and after applying a stress annealing treatment performed at 700 °C at a pressure of 3.7 × 10^−7^ Pa for 2 h. The magnetization process and magnetic domain dynamics were evidenced at the edge and center of the core tooth based on magnetic field application parallel to the rolling direction (RD) of the material. The Kerr images showed that the grains placed near the cut edge had a stripe domain configuration, which ran in a direction perpendicular to RD. In addition, the magnetization vector was identified to be parallel to RD. The grains placed at the tooth center were characterized by a complicated domain pattern and stripe domains parallel to RD. The authors concluded that the mechanical cutting procedure did not influence the central region of the samples because the magnetic domain structure was similar to that of rolled NO FeSi. It was proven that the magnetic domains were modified only in the cut-edge neighborhood due to punch-induced stresses. After the annealing treatment application, it was noticed that some stripe domain patterns placed parallel to the transverse direction (TD) changed in the stripe domain perpendicular to TD near the cut edge. This modification was attributed to the stress relief effect of the treatment. The main conclusion of this study was that the shear stress induced by the punching procedure changed the domain structure near the cut vicinity. Naumosky et al. [64] made a magneto-optical evaluation of the punching influence on magnetic properties of fully processed NO FeSi with three alloying contents (Si + Al wt.% ranging in 1:2.5:3.2) and three different grain sizes (1:2:4). All the investigated samples had a thickness of 0.35 mm. The authors’ MOKE study was made only to show the magnetic domains that were not pinned due to the punching effect. It was put in evidence that near the punched edge, a hardened zone of about 220 μm with no magnetic contrast appeared. These results were correlated with hardness measurements and proved the presence of dislocation. The authors concluded that dislocations generated by punching are the main process that induced the pinning of the domain walls, leading to a magnetic hardening effect. This magnetic hardened zone was estimated to be three to four times deeper into the material structure compared to the mechanical hardened zone. The samples with the highest alloying content and the biggest grain sizes were the most affected by punching. The main conclusion of this study was that the quantitative evaluation of the local magnetic contrast from MOKE images can provide important information about the size and shape of the magnetically affected zone near the cut edge.

**Figure 5:**
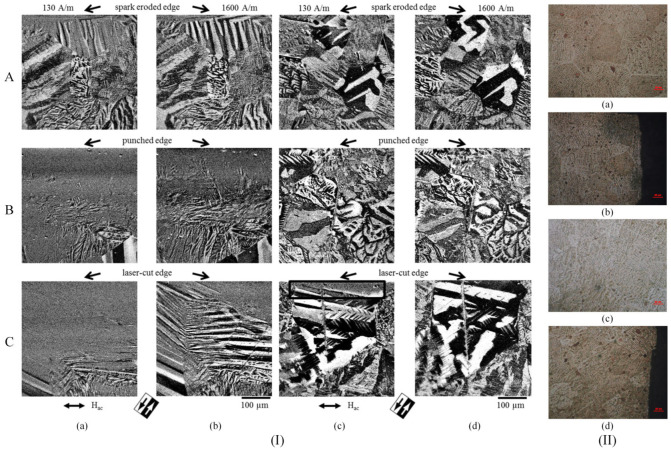
MOKE and Bitter micrographs of NO FeSi alloys: (**I**) Kerr images of magnetization distributions near the cut edge obtained through (**A**) spark erosion, (**B**) punching, and (**C**) laser cutting at two values of the applied magnetic field: (**a**,**c**) for 130 A/m, (**b**,**d**) for 1600 A/m ((**a**,**b**) were considered before SRA, while (**c**,**d**) were taken after SRA) [40]. Reprinted from [40] Copyright (2024), with permission from Elsevier; (**II**) Magnetic domain images obtained by Bitter technique on (**a**,**c**) un-damaged and (**b**,**d**) cut-edge zones of samples cut through laser method in the case of (**a**,**b**) 7.70 g/cm^3^ and (**c**,**d**) 7.80 g/cm^3^ alloys densities [65]. Figure is licensed under CC—by 4.0.

Hofmann et al. [41] evaluated the microscopic impact of laser, spark erosion, and guillotine cutting as well as the effect of SRA treatment based on MOKE. They intended to determine the magnetic softness in the cut-edge vicinity by combining the classical MOKE approach with the difference image technique. The authors could observe only the domains that were changed while applying an external magnetic field. It was noticed that the zone affected by spark erosion is placed in a small area near the cut edge. The grains situated in this zone have a degraded magnetization behavior. On the other hand, a fine striped domain pattern parallel to the sample plane was evidenced in the case of guillotine cutting. It was concluded that the magnetic properties near the cut edge are changed due to dislocations and plastic deformations, exhibiting a much larger zone (about 700 μm) than spark erosion. For laser-cut samples, the domain pattern contained long, wide strips of parts that looked distorted. The authors observed that the deterioration of the domain pattern is higher than in the case of mechanically cut samples, which can increase the magnetic losses of the material. After the SRA treatment was applied, only the laser cut samples exhibited a degradation of magnetic behavior, while for the other two cutting technologies, a healing of the micromagnetic properties was noticed. The images were characterized by an improved contrast over the investigated zone. Naumoski et al. [40] prepared samples with 2.8 wt.% Si, average grain size of 96 μm, and thickness of 0.35 mm based on laser and punching technologies and compared the obtained results with those measured on a reference sample cut through electroerosion. In the case of punched samples, a degraded zone of about 100–150 μm was put in evidence based on MOKE images. For the laser cut samples, a distorted domain pattern exhibiting wide and long stripe domains was seen combined with a zone with deteriorated magnetic properties of about 300 μm, while in the case of electroeroded samples, the degradation of the magnetic properties was present in a narrow region of under 100 μm (Figure 5I).

Another classical and low-cost method used to observe the magnetic domain was developed by Bitter F. [66] and consisted of magnetic nanoparticle fluid use. Cao et al. [7] investigated the effect of the punched stress on the magnetic domain structure of 50WW470 NO FeSi samples produced by WISCO based on the abovementioned method. They prepared Fe_3_O_4_ nano-magnetic fluid using the coprecipitation method. The Bitter images revealed that in the case of as-cut samples, a complicated domain pattern emerged, including 180°-domain, 90°-domain, and wave-domain. The cut edge contained many closure domains that decreased the magnetostatic energy, and only a few 180°-domains were noticed on the sample edge. The domain structure changes could be observed only within the area of 0.3 mm, comprised between the edge and sample center. After an annealing treatment was applied, a highly ordered domain structure was evidenced. Many domains had stripe-like shapes, with 180° and 90° domain walls. It was noticed that the magnetic domain structure found near the cut edge was similar to that present in the sample center, except for a minor change in the domain structure identified across a small area found at 80 μm from the edge to the center. The main conclusion of the study was that a stress relief annealing is usually recommended to remove the unwanted effects of cutting. Xiang et al. [65] analyzed the influence of the laser parameters in the case of six types of electrical steel laboratory prepared with a mass density comprised between 7.60 and 7.85 g/cm^3^ cut through laser technology. They visualized the magnetic domain structure for two types of NO FeSi (with a mass density of 7.70 and 7.80 g/cm^3^) based on Bitter method and prepared Fe_3_O_4_ magnetic fluid (Figure 5II). It was observed that the parallel strips are associated with the magnetic domains, and near the cut edge, the Bitter method showed the existence of domains strongly altered by the laser technology. The authors concluded that these zones could determine a decrease in the total losses. In addition, the width of the magnetic domain decreased directly proportional to the Si content, which varied from 0.971 to 0.617 wt.%. The main conclusion of the paper consisted of underlining the importance of a machine learning algorithm that can control the laser parameters to adjust the losses and other material properties.

Magnetic domain structure investigation provides a link between cutting technology and the magnetization vector of the materials. Based on this method, one can observe and practically see and measure the shape and size of the affected area near the cut edge and even estimate its geometrical dimensions. In addition, the evolution of magnetization can be investigated under a magnetic field effect. In this way, the main differences between the damaged zone by cutting and the unaffected area can be evidenced. The stress relief annealing treatment generates modification in the magnetic domain structure of NO FeSi, and the Bitter or MOKE method can be chosen as an adequate tool to investigate these phenomena.

## 4. Influence of Cutting Technology on Magnetic Properties of Non-Oriented Electrical Steels

The magnetization process analysis in NO FeSi is of the utmost importance in the improvement of electric motor performance, which depends on efficient energy conversion [46,67]. The cutting procedure is today considered a critical step in core production due to the fact that induced mechanical or thermal stresses deteriorate the soft magnetic response of the electrical steel, as presented in Section 3. In this paper section, the influence of cutting technology in a direct relationship with the quasi-static and industrial frequency behaviors of the investigated material will be described. That consists of presenting normal magnetization curves and quasi-static energy losses reported by different research studies, dynamic loss evolution, as well as analytical and numerical models, which took into consideration the cutting technology effect on different magnetic physical quantities. The main devices that characterize NO FeSi are the single sheet/strip tester (SST), the Epstein frame, and magnetic permeameters that can perform measurements in alternating or direct current fields.

### 4.1. Experimental Investigations

#### 4.1.1. Normal Magnetization Curves and Quasi-Static Energy Losses

The normal magnetization curve is usually constructed in an alternating current (AC) magnetic field, being defined as the geometrical place of the maximum values of the pair (*H*, *B* or *M* or *J*) extracted from the symmetric about the origin hysteresis cycles, evolving from the demagnetized state to saturation (*B*—magnetic flux density, *M*—magnetization and *J*—magnetic polarization). The normal magnetization curve differs from the initial magnetization curve, which is constructed under the effect of a direct current (DC) magnetic field applied starting from a demagnetized state or from the virgin magnetization curve that requires heat treatment at a temperature higher than the Curie point of the ferromagnetic material and cooling in a zero-field environment [5,10,46]. The hysteresis energy losses are usually computed or measured in quasi-static conditions, being an adequate indicator of the magnetic property changes, enabling the control and improvement in energy consumption of electric motors, as well as developing innovative materials in accordance with the latest efficiency regulations [68].

Weiss et al. [6] assessed the deterioration of magnetic properties induced by the shearing procedure. To that goal, lamination samples with an area of 120 mm × 120 mm from NO FeSi with a thickness of 0.35 mm and 0.5 mm were prepared. The magnetic characterization was performed with a standardized SST. To analyze in detail the effect of mechanical cutting, they increased the cutting perimeter by preparing strips of different widths (2 × 60 mm, 4 × 30 mm, 8 × 15 mm, 12 × 10 mm, 24 × 5 mm) and by putting them alongside to obtain the standard width of 120 mm. In their study, they used two types of tools, one sharp and another worn. It was noticed that the magnetic behavior of the electrical steel deteriorates directly proportional to the increasing number of cutting lines. By analyzing the normal magnetization curves, it can be concluded that within the range between 0.5 T and 1.5 T, cutting influence is very important, whereas near saturation, this effect is significantly diminished. All the normal curves are expected to meet at the same final point in the high-induction domain. The authors concluded that the type of cutting tool and cutting perimeter increase are two important factors that must be considered when magnetic cores of high-efficiency electrical motors are manufactured. Füzer et al. [39] determined the clearance of the cutting tool influence on the quasi-static properties of M350-50A ring samples with inner and outer radii of about 15 mm and 25 mm in the un-annealed and annealed state. The cutting tool clearance was set at 1%, 3%, 5%, and 7% of the electrical steel thickness. The authors determined the hysteresis energy losses *W*_h_ under quasi-DC conditions, providing an average value of about 240 J/m^3^ for untreated and about 130 J/m^3^ for annealed samples. The corresponding minimum values of the *W*_h_ were reported for clearance values of 5% and 3%, respectively. It was stated that the clearance of 5% in the case of untreated samples had the lowest damaging effect on the electrical steel structure induced by deformation stresses accountable to supplementary pinning site apparition that hindered the domain wall displacement.

**Figure 6:**
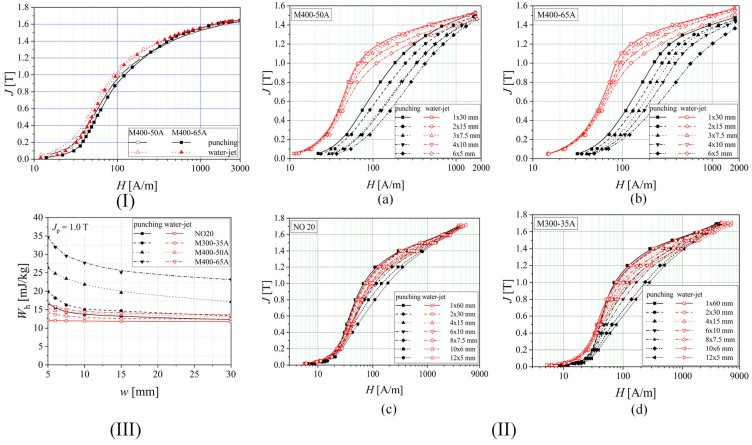
Experimental investigations of quasi-static behavior for different grades of NO FeSi alloys: (**I**) Comparison between normal magnetization curves of M400 grade steel having different thickness cut through punching and abrasive water-jet methods for a 30 mm × 300 mm sample area; (**II**) Examples of 2 Hz quasi-static determinations on samples cut through mechanical punching and abrasive water-jet technologies: normal magnetization curves for (**a**) M400-50A, (**b**) M400-65A, (**c**) NO20 and (**d**) M300-35A electrical steel grades; (**III**) Hysteresis energy loss variation as a function of strip width [35]. Figure is licensed under CC—by 4.0.

Hofmann et al. [41] presented the influence of laser, mechanical cutting, and spark erosion technologies on fully processed non-oriented electrical steel normal magnetization curves experimentally measured with an SST. Firstly, the authors cut, through spark erosion, an M330-35A electrical steel sample with an area of 150 mm × 150 mm parallel to RD, which was used as a reference. Then, they cut through laser, guillotine, and spark erosion a number of strips with variable widths of 4, 6, 8, 10, and 30 mm and combined them to obtain the reference specimen width (150 mm) and multiply the effect of the cutting. The normal magnetization curves showed that all the technologies induced a degradation in the magnetization process, which was more pronounced below the magnetic flux density threshold of 1.5 T. The laser procedure determined the apparition of a larger damaged zone due to a rapid heating change in magnetic properties and metallurgical characteristics. Mechanical cutting generated a much-reduced effect compared to laser. At the same time, the spark erosion procedure led to a magnetic behavior comparable to that of the reference sample for the whole range of measurements. It was concluded that the laser technology damaged the magnetic properties in the low induction range in a high amount but became superior to the guillotine at inductions above 1.2 T, denoting a better magnetization process. It was concluded that the laser technology damaged in a high amount the magnetic properties in the low induction range but became superior to the guillotine at inductions above 1.2 T, denoting a better magnetization process. It can be observed that the mechanical stresses induced in the high-induction domain are much stronger than the heat stresses generated by the laser procedure. The severity of the degradation process evolved from spark erosion to mechanical procedure and laser technology. Shi et al. [52] performed a similar research, which supplementarily included the abrasive WJ technology. They prepared samples in the classical shape (30 mm × 30 mm) and measured the magnetic properties based on the Epstein frame. In addition, they investigated the effect of SRA. After the normal magnetization curves were determined at a frequency of 50 Hz, the authors noticed that the magnetic flux density exhibited the lowest value in the case of laser technology, followed by punching and wire-EDM. That allowed the authors to conclude that the abrasive WJ technology led to the best magnetic evolution by damaging the material magnetic properties in a low amount. After the SRA application, all the normal curves overlapped, demonstrating once again the importance of annealing treatment in removing the residual stress due to cutting procedures and its healing effect on the metallurgical characteristics. The authors recommended the abrasive-WJ as the most non-invasive method but also underlined that a reduced processing speed characterizes this technology. Naumoski et al. [40] prepared rings made from 0.35 mm thick NO FeSi with an inner diameter of 45 mm and an outer diameter of 55 mm based on electroerosion, punching, and laser technologies, respectively. The authors measured the initial magnetization curves by applying a DC magnetic field. They found that the EDM was directly linked to an almost ideal magnetization process, followed by punching and laser methods. Regarding the latter, a shift at 1000 A/m magnetic field strength was reported. Above this value, laser technology becomes better than the mechanical method, but below this point, the laser-cut sample magnetization process slowly evolves in a linear manner characterized by high magnetic fields and low inductions in comparison with punching. The authors concluded that the laser technology led to the most difficult magnetization process in ring samples and that the thermal stresses manifested an increased effect of damaging the material magnetic structure. Schoppa et al. [26] investigated the influence of abrasive WJ compared to laser and mechanical cut samples. They cut samples with a length of 160 mm and variable width of 30, 15, 10, 7.5, 6, and 5 mm and put them together to obtain a width of 30 mm. The material magnetic characterization was performed on an SST, and its main purpose was to emphasize the positive effect of WJ in comparison with the other two methods since their negative influence on NO FeSi magnetic behavior was already proven by other similar conducted research [5,11,32,46]. Paltanea et al. [35] investigated the magnetic behavior of four types of electrical steels (M400-50A, M400-65A, NO20, and M300-35A) cut through mechanical punching and abrasive WJ (Figure 6I). For the experimental measurements, two SST setups were used, each having a 300 mm length and widths of 30 mm and 60 mm, respectively. The samples were prepared by cutting a different number of strips with variable widths of 5, 6, 7.5, 10, 15, 30, and 60 mm to assemble a standardized strip of 30 mm × 300 mm for M400-50A and M400-65A industrial grades and 60 mm × 300 mm for the other two high-quality steels. Figure 6II showed that by decreasing the strip width, a fact that determines the increase in the cutting perimeter, the magnetization process became more difficult for both technologies. It can be seen that this effect is much more pronounced in the case of the punching method. By comparing the results obtained, it can be seen that for the thinner materials (NO20 and M300-35A), the differences between the impact of the two cutting technologies are reduced compared to the thicker alloys. The authors computed the hysteresis energy losses by extrapolating to zero the experimentally determined total energy losses as a function of frequency (Figure 6III). The maximum values were obtained in the case of 5 mm width samples for all the investigated materials with prevalent effect given by the punching (e.g., M400-65A 5 mm width: punching—*W*_h_ = 35 mJ/kg, abrasive WJ—*W*_h_ = 17 mJ/kg). Minimum values of hysteresis losses were noticed for 60 mm width WJ cut NO20 samples (e.g., NO20 60 mm width: punching—*W*_h_ = 15 mJ/kg, abrasive WJ—*W*_h_ = 12 mJ/kg). The authors concluded that the abrasive WJ technology provides the four NO FeSi with the best-preserved quasi-static magnetic behavior, having a minimum detrimental effect on material properties.

The normal magnetization curves fully describe the magnetic material behavior, providing a clear view of the magnetization processes that occur in NO FeSi, mainly composed of domain wall moments. In contrast, uniform magnetization rotations inside the magnetic domains only appear at the saturation point. The quasi-static evolution of the magnetic material is noticed only when the magnetization rate of change is reduced, so dynamic viscous-type effects do not combine with the magnetization processes. One can observe that the point-by-point determination of the normal magnetization curve is a very facile procedure consisting of measuring symmetric hysteresis loops and then recovering their tip points. The cutting technology exhibits important influences on the material magnetization processes, a fact that can be seen when thermal or mechanical stresses are induced. The magnetization process becomes much more difficult concomitantly with an increase in the hysteresis cycle area, which is usually used to compute the hysteresis energy losses. Investigating the material quasi-static behavior is important because it allows for calculating the hysteresis losses, which the electric motor manufacturers usually consider an important part of the iron losses that must be reduced to comply with newly introduced high-efficiency regulations.

#### 4.1.2. Total Losses and Dynamic Behavior

In the case of a rapid magnetization rate of change, the so-called rate-dependent hysteresis phenomena are present. For metallic ferromagnetic materials, they are generated in a high amount due to eddy currents, whose circulation in the samples is a function of magnetization rate, electrical resistivity of the material, magnetic domain structure, and geometrical dimensions and shape of the samples. It must also be mentioned that the important consequences of the viscous effects are combined with long-range eddy currents, determining an increase in the total energy dissipated during the magnetization process. This phenomenon is characterized by a broadening of the hysteresis cycle when the measuring frequency increases [46,69]. In this section, some examples of how the cutting procedure influences the total power or energy losses are provided, as well as some studies that computed the dynamic losses with their two components: the macroscopic one due to eddy currents occurring along circular patterns inside the material (usually termed as “classical losses”) and another one characteristic for microscopic level, which is due to the micro eddy currents that flow within the domain wall, to be found in the literature as “excess losses”.

Weiss et al. [6] measured the specific power losses at 50, 100, 400, and 750 Hz on mechanically cut samples utilizing worn and sharp tools, respectively. The increase in cutting perimeter determined a much-pronounced variation in the specific losses with values between 3 W/kg (120 mm width sample taken as reference, sharp and worn tools at 0.15 m/s) and 4.9 W/kg (5 mm width sample, sharp tool at 0.15 m/s). On the other hand, the authors noticed that the influence of tool characteristics or cutting speed exhibited a reduced impact on loss variation (e.g., for 0.5 mm thick NO FeSi, 5 mm width strip, at 100 Hz: worn tool/0.04 m/s–4.8 W/kg, worn tool/0.15 m/s–4.9 W/kg; sharp tool/0.04 m/s–4.4 W/kg, sharp tool/0.15 m/s–4.5 W/kg). The dynamic analysis at different frequencies showed that the lowest power loss values were obtained in the 120 mm width sample case, which was not influenced by the cutting parameters. The dynamic analysis at different frequencies showed that the lowest values of power losses were obtained in the case of the 120 mm width sample, which was not influenced by the cutting parameters. For the 5 mm width strip, normalized specific losses rated with the reference sample were 1.33 at 50 Hz, 1.31 at 100 Hz, 1.25 at 400 Hz, and 1.22 at 750 Hz. The authors concluded that in order to analyze the power losses, many more cutting parameters must be considered.

**Figure 7:**
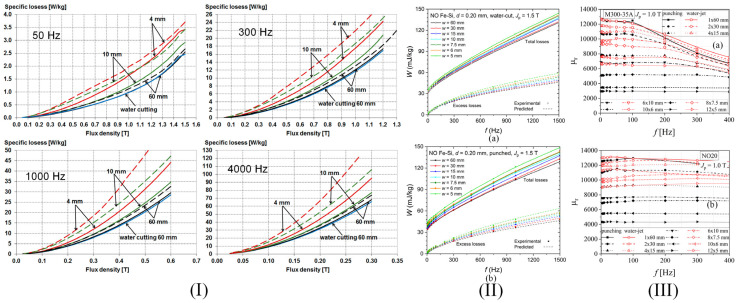
Experimental and computed dynamic behavior of different grades of NO FeSi alloy samples cut at different widths: (**I**) Specific power loss variations of a M270-35A grade steel cut through punching (solid line) and laser (dashed line) methods at different excitation frequencies [70]. Figure is licensed under CC—by 4.0; (**II**) Total energy and excess energy losses versus frequency at 1.5 T in the case of a Cogent NO20 Hi-Lite steel grade samples cut through (**a**) water-jet and (**b**) punching [5]. Reprinted from [5] Copyright (2024), with permission from Elsevier; (**III**) Relative permeability versus frequency in the case of (**a**) M300-35A and (**b**) NO20 steel grade samples cut by punching and water-jet technologies [35]. Figure is licensed under CC—by 4.0.

Hofmann et al. [41] measured the magnetic power losses under sinusoidal magnetic flux density waveforms at 50 Hz by variating the induction amplitude from 0.2 to 1.8 T. A general conclusion that was observed in the case of all the cutting technologies was that by increasing the cutting perimeter, a direct proportional variation in power losses was proven. The following examples were given in their study: in the case of 6 mm width strips, a pronounced power loss increase of about four times higher occurred in the low induction range, while about 1.5 times loss increase was detected for magnetic flux densities higher than 1.5 T, in comparison with the 150 mm width strip considered as reference; the mechanical cutting was characterized by a loss increase between 30% and 70%, while the spark erosion led to an increase in the range of 5% to 22%. Shi et al. [52] investigated the effect of cutting methods on power losses and magnetic permeability of NO FeSi at the industrial frequency of 50 Hz. They evidenced that before the SRA treatment application, the experimentally measured iron losses of laser and punched cut samples had a maximum value of about 3 W/kg, followed by the shearing procedure (2.7 W/kg), and wire-EDM and abrasive WJ characterized by a value of about 2.5 W/kg. After the SRA application, it was noticed that the value of power losses is almost the same (2.6 W/kg). The relative magnetic permeability of the electrical steel was highly influenced by the cutting procedure, with differences of about three times between laser cutting (maximum value of 4000) and abrasive WJ (maximum value of 11,500). It was concluded that the laser method damaged the steel magnetic properties more pronounced due to induced thermal stresses, although this procedure had a positive effect on grain growth in the cut edge region, a fact usually considered beneficial for magnetic properties. After the SRA treatment, the healing effect was obvious in the case of relative magnetic permeability with low variations between 12,000 (abrasive WJ) and 14,000 (laser). Naumoski et al. [40] determined the absolute loss values measured at 50 Hz/1.5 T and 400 Hz/1 T. The highest values of about 2.86 and 22.8 W/kg were obtained for the laser-cut samples, respectively. For the other two methods, the authors measured 2.78 and 21.8 W/kg in the case of punching and 2.75 and 21.6 W/kg for the spark erosion technology. Dems et al. [70] measured the specific power losses on M270-35A electrical steel cut through the guillotine and laser. There were characterized sheets with an area of 300 mm × 60 mm with an SST. The 60 mm width was obtained by putting together a number of strips between 1 and 15 with variable widths in the range of 4–60 mm. Also, there were prepared samples cut at an angle of 0°, 30°, 45°, 60°, and 90° with the rolling direction. In Figure 7I are presented the selected results of specific power losses versus magnetic flux density at different frequency values in the case of laser and punching methods in comparison with the results obtained for a waterjet cut reference sample with a width of 60 mm. It can be noticed that for 4 mm width samples mechanically cut, the loss value normalized to the reference sample exhibited an increase of about 50%, since for the laser-cut, the increase was estimated at 100%. On all the chosen frequency values, the differences mentioned above decreased with the width increase. The authors concluded that at 60 mm strip width, the loss increase determined for the punching method was of the order of a few percent and no more than 15% in the case of laser cut samples.

The concept of loss separation into three components, hysteresis, classical, and excess losses, was applied in some studies to better understand the influence of the cutting procedure on the dynamic behavior of NO FeSi. Naumoski et al. [64] prepared ring samples of 0.35 mm thickness having different grain sizes and Si + Al alloying contents through EDM (reference sample) and punching and characterized them in the same conditions as in [40]. The classical losses were analytically computed and considered unaffected by the cutting procedure at given frequencies and magnetic flux density values. In the case of high Si + Al content and varying the grain size from big to small, the quasi-static hysteresis losses increased (e.g., at 400 Hz/1 T: big-grained material: 5 W/kg; small-grained material: 10 W/kg), while the excess losses decreased (e.g., at 400 Hz/1 T: big-grained material: 11.5 W/kg; small-grained material: 6 W/kg). It was noticed for the samples with small size grains that the hysteresis losses were prevalent, and in the case of big-grained alloys, the excess losses were the dominant component. The authors showed that for 50 Hz/1.5 T conditions, the hysteresis losses had the most important contribution, increasing from 1.35 W/kg (big-grained material) to 2.6 W/kg (small-grained material). Regarding the excess losses, a decrease between 1.05 W/kg (big-grained material) and 0.6 W/kg (small-grained material) was achieved. It was concluded that the grain size affected the degradation of magnetic properties due to punching in a high amount, and in the case of big grains, the magnetic hardened zone became larger. Manescu (Paltanea) et al. [5] investigated the influence of mechanical and waterjet cutting on the dynamic magnetic behavior of Cogent NO20 Hi-Lite and M300-35A electrical steels with grain sizes of 122 μm and 86 μm, respectively. They prepared samples with different widths between 5 and 60 mm and a length of 300 mm. The magnetic measurements were made in accordance with the existing standard EN IEC 60404-3:2022 [71] by mixing RD and TD cut strips to investigate if the 10–15% magnetic anisotropy modified the values of energy losses. It was demonstrated that the sheet cutting direction exhibited a negligible effect on the strain-hardening phenomenon. The authors placed a number of 12 to 1 strips side-by-side to obtain 60 mm width samples. The energy losses versus frequency *W*(*f*) were experimentally measured with the help of a laboratory digital wattmeter at the peak polarizations (*J*_p_) of 1.0 T and 1.5 T with a maximum frequency of 1500 Hz and 400 Hz, respectively. The authors computed the classical energy losses (*W*_cl_) by assuming that this loss component is associated with eddy currents present at the macroscopic level and with the absence of skin effect, being a function of strip resistivity, thickness, and density, and directly proportional to the frequency at a given value of *J*_p_ [35,72]. Reference [35] reported maximum values of *W*_cl_ at *J*_p_ = 1 T and *f* = 400 Hz of about 6 mJ/kg (NO20 grade) and 20 mJ/kg (M300-35A). The excess energy losses (*W*_exc_) were calculated by subtracting the sum of hysteresis and classical losses from the total losses. The results were compared to those obtained after applying the Statistical Theory of Losses [73] as seen in Figure 7II [5]. This framework developed by Bertotti et al. [74] took into account through its statistical parameters, the local coercivities and the discrete nature of the magnetization process. After analyzing the loss behavior for both types of electrical steels, it was noticed that hysteresis and excess losses increase directly proportionally with the cutting perimeter due to the magnetic hardness effect generated by mechanical punching. The energy losses obtained for the waterjet cut samples are lower than those measured for the mechanically cut ones, as described by Paltanea et al. [35], which is a complementary study to the analysis presented in [5]. Regarding the relative magnetic permeability, it was concluded that the waterjet method exhibited a small influence in comparison with the punching technology, as can be observed from Figure 7III. The lowest values were noticed for the 5 mm width strips at 400 Hz/1 T conditions, as follows: for punching, 4400 (NO20 grade) and 3000 (M300-35A); for water, 9500 (NO20 grade) and 5500 (M300-35A). The authors concluded that despite the drawbacks of abrasive WJ technology, it preserved the best magnetic properties of the investigated NO FeSi in terms of energy losses and relative magnetic permeability and should be considered when special motors with high efficiency are manufactured, while punching must be used in the mass production of high-quality electrical motors due to the fact that it influences in a high amount the magnetic behavior of the material. Füzer et al. [39] measured the energy losses of M350-50A mechanically cut ring samples by modifying the tool clearance. A digital standardized hysteresisgraph was used to provide magnetic quantities values at a peak magnetic flux density of 1 T up to a frequency of 400 Hz. It was noticed that for the non-annealed samples, the minimum value of energy losses was determined for the cutting tool with 3% clearance at frequencies lower than 300 Hz, while for upper frequencies, the samples cut with 1% clearance had the best magnetic behavior. In the case of annealed samples, the 3% clearance cutting tool was linked to an important loss suppression for all the frequency ranges, and the 5% clearance condition sample did not show any improvements in comparison with the loss variation obtained for the non-annealed state. The authors performed the loss separation described in [72] and concluded that only the quasi-static hysteresis and dynamic excess losses are influenced by the cutting procedure, while the classical losses are unaffected by it as described in many studies [5,35,40]. Based on magnetic objects theory [74] and statistical theory of losses [46], they found that *W*_exc_ is the most sensitive component to the cutting clearance influence, and their magneto-structural relationship is characterized by the uniform pinning center and field distribution in the non-annealed state, evolving to a non-uniform distribution after the annealing treatment.

Investigating the total losses and the dynamic behavior is critical at the development stage of high-efficiency motors, considering that these types of devices are submitted to AC magnetic fields and controlled by variable frequency drives. Summarizing, as a direct consequence, the analysis of each loss component and its variation with the frequency and peak magnetic flux density can provide the producers with important insights into how the final product will perform in different conditions, as well as how to change the material attributes to keep the losses in a given range of limits [75,76]. In addition, the way cutting technologies influence the loss of components can be applied to choose a proper method to manufacture certain parts of the motor magnetic cores and to better understand the microstructural changes that occur during processing steps.

### 4.2. Analytical and Numerical Models

Considering the cutting process’s influence on the magnetic properties of the strips that make up the magnetic cores is of the utmost importance in electrical motor production. In the case of small motors, the deteriorated zone width becomes a parameter that must be assessed and implemented in analytical motor models. Indeed, as previously discussed, the damage zone’s extent is directly linked to critical parameters in the design, such as the relative magnetic permeability, total hysteresis, excess power or energy losses, and normal magnetization curves. The simplest models in the literature consider that lamination has two main parts: the central one characterized by undamaged magnetic behavior or properties and other small zones placed near the cut edge, strongly affected by the cutting procedure. This problem is extensively investigated in some review papers [20,77], while the most intuitive and easy-to-implement models are presented in [5,10,78,79].

Studies [5,10] developed an innovative model that considers an invariable width of the damaged zone by using different width samples to model the effect of the cutting procedure. Another model permitted the computation of loss figure and average value of magnetization based on experimental measurements performed on only two strips of different widths, allowing the determination of magnetic flux density values in the unaffected and damaged zones by the cutting procedure [79,80]. Third-order spline functions were used to obtain the magnetization vector behavior outside the measuring points, with increased attention to the Rayleigh zone mapping and saturation region analysis. By reading many analytical formulations in the literature, it can be inferred that the concept of electromagnetic parameters’ variation as a function of the cut edge extent is the most frequently applied. The study presented in [81] developed a unique approach consisting of cut-out stator slots with windings placed in the slots to concentrate the magnetic flux in the teeth area. The mathematical basis of the investigation assumed that magnetic permeability variates exponentially, with a pronounced decrease from the tooth central zone toward the cut edge. In addition, a simple relationship for core losses as a function of magnetic flux density was provided. The variation in the magnetic flux density versus the grain size and Si weight percent was presented in [82]. This model was very interesting, but unfortunately, the chaotic changes of the parameters did not permit further steps in the direction of the development of a link with the degradation of magnetic quantities. Another model that considered the variation in the magnetic permeability with the cut edge distance used a parabolic function, which included both the cutting characteristics and material parameters [83] and offered a polynomial expression of the loss density distribution. Papers [84,85] provided a variation in magnetic permeability based on an exponential function of the magnetic field strength and a simple formula for the power loss computation by assuming that the cutting procedure only influences the hysteresis loss component. Paper [86] expressed permeability as a function of an inverse tangent based on magnetic measurements performed in two points, while in studies [87,88], an exponential degradation profile for the magnetic permeability was adopted, and a formula useful to compute the hysteresis and eddy current losses was developed by considering that these two components are both affected by the cutting process. An innovative approach was developed in [89], presenting a variation in the magnetization vector based on the Langevin function that permitted the authors to develop a theory by including the Gumbel distribution to provide an analytical expression of damaged steel magnetic susceptibility. Table 4 summarizes the main analytical models reported in the literature [80,81,82,83,84,85,86,87,88,89,90,91,92,93,94].

**Figure 8:**
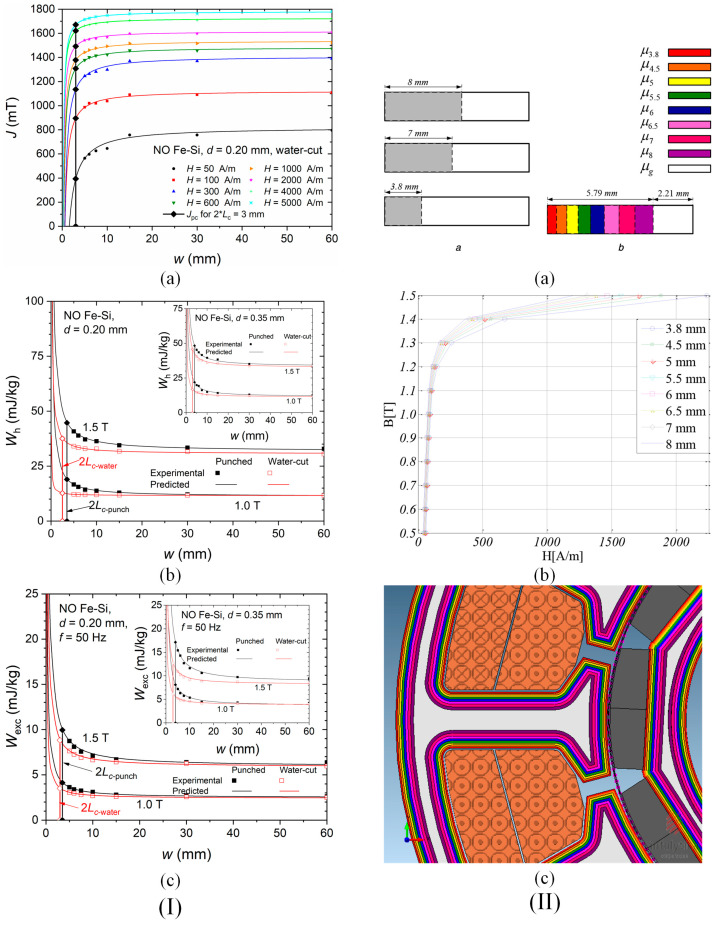
Analytical and FEM approaches to include the influence of cutting technology on the magnetic properties variation in NO FeSi alloys: (**I**) Hyperbolic model to compute the magnetic polarization dependence as a function of the strip width: (**a**) experimental measurements to determine the model parameters, and comparisons between measured and predicted values of the (**b**) hysteresis and (**c**) excess energy losses of NO20 and M300-35A (inset) grades [5]. Reprinted from [5] Copyright (2024), with permission from Elsevier; (**II**) 2D FEM of a PMSM that uses a multi-layered model: (**a**) exemplification of the 1 to 8 layers having different relative permeabilities values as a function of the distance from the cut-edge, (**b**) Computed B(H) dependences, and (**c**) software implementation of the layered model for the stator and rotor laminations [95]. Reprinted from [95] Copyright (2024), with permission from © 2020 The Institution of Engineering and Technology.

In this section, there will be presented, besides the analytical approach of cutting technology, the most important numerical simulations based on the finite element method (FEM), which discuss the interaction between mechanical properties related to punching or shearing and the modification of magnetic properties. These aspects are worth mentioning because the manufacturing effect must always be considered before a high-efficiency motor is designed, having to comply with predefined characteristics and specific applications. In Table 4, some interesting approaches that were based on electromagnetic simulations in commercial programs or in-house developed software [80,84,86,89,90,92,93,94,95,96,97,98,99,100,101,102] are introduced. In Figure 8 are presented analytical and FEM approaches that include the cutting technology effect on magnetic properties variations of NO FeSi.

**Table 4:** Analytical and numerical models of cutting technology influence on electromagnetic behavior of NO FeSi.

**Analytical Models** (*d* = index denoting degraded property due to the cutting procedure, *n* = index denoting non-degraded property)				
**Analyzed Physical Quantities**	**NO FeSi Type/Cutting Technology**	**Analytical Formulation**	**Remarks**	**Ref.**
Relative magnetic permeability μ, power losses *p*_Fe_	M330-50A/punching	$\mu \left(H,x\right)=\mu_{n}\left(H\right)\left(1-\frac{2}{\pi }\mu_{p}e^{\frac{-x}{a\mu }}\left(\arctan\left(\frac{H}{H_{1}}\right)-\arctan\left(\frac{H}{H_{2}}\right)\right)\right),$ $P_{Fe}\left(B,x\right)=k_{h0}\left(1+k_{p}e^{\frac{-x}{a_{p}}}\right)fB^{\alpha_{0}}+k_{e}\left(1+x_{10}B^{x_{20}}\right)f^{2}B^{2}$	- The exponential function models the decrease in the cutting effect with the increase in the distance from the cut edge; - The magnetic field strength terms are related to imposing a limitation of the cutting effect in the zone beneath and around the normal magnetization knee region	[86]
μ, *p*_Fe_	M330-50A/punching and EDM	$\begin{array}{l} \mu_{r,d}\left(H\right)=\mu_{r,n}\left(H\right)\left(1-e^{\frac{-H}{\tau }}\right)+1,\tau \left(x\right)=\frac{\gamma }{1-{\left(\frac{x}{d}\right)}^{c}},\\ p_{Fe}=\left(k_{d}x+q\right)k_{h}{\left(\frac{B}{B_{0}}\right)}^{n}\left(\frac{f}{f_{0}}\right)+k_{ec}{\left(\frac{B}{B_{0}}\right)}^{2}{\left(\frac{f}{f_{0}}\right)}^{2} \end{array}$	- Variable *x* represents the distance from the cut edge, while *d* is the width of the damaged zone; - *B*_0_ = 1.5 T and *f*_0_ = 50 Hz; - The authors assume that only the hysteresis losses are influenced by the cutting; - The damaged width zone was considered 1 mm	[84,85]
Magnetic flux density *B*, magnetic field strength *H*	-	(*B*_i_, *H*_i_) is mathematically scaled to (*B*_i_, *H_i_*/γ(s)), $\begin{array}{l} \gamma \left(s\right)=1-\left(1-\hat{\gamma }\right)e^{\frac{-s}{\delta_{s}}},\\ \sigma_{hist}^{*}=\frac{\delta_{hist}}{\gamma \left(s\right)} \end{array}$	- Parameter $\hat{\gamma }$ is the degradation factor near the cut edge, *s* represents the distance from the cut edge, δ_s_ is considered the depth at the degradation effect is reduced to one-third; - all these parameters are dependent on experimental data available in the literature	[90]
*B*	M250-50A, M400-50A, M800-50A, M940-50A/punching	$B_{\max}\left(T\right)=\frac{a}{{\left(1+e^{b-cx}\right)}^{\frac{1}{d}}}$	- The parameters *a*, *b*, *c*, and *d* are a function of grain size and Si content; - Up to 2.5 wt.% Si content, the model parameters had low variations, while above this limit, an important variation was noticed	[82]
Normal magnetization curve *J*(*H*), peak magnetic polarization *J*_p_ hysteresis *W*_h_ and excess energy *W*_exc_ losses	NO20, M300-35A/punching and WJ	$\begin{array}{l} J_{p}\left(w\right)=J_{p,n}-\left(J_{p,n}-J_{p,d}\right)\frac{2L_{c}}{w};\\ W_{h}\left(w,J_{p}\right)=W_{h,n}\left(J_{p,n}\right)+\left(W_{h,d}\left(J_{p,d}\right)-W_{h,n}\left(J_{p,n}\right)\right)\frac{2L_{c}}{w},\\ W_{exc}\left(w,J_{p},f\right)=W_{exc,n}\left(J_{p,n},f\right)+\left(W_{exc,d}\left(J_{p,d},f\right)-W_{exc,n}\left(J_{p,n},f\right)\right)\frac{2L_{c}}{w} \end{array}$	- Parameter *L*_c_ represents the width of the damaged zone, *w* is strip width (5–60 mm), and *f* is the working frequency; - The *L*_c_ parameter is estimated by analyzing the exponential decrease in *J* as a function of the strip width; - Only the *W*_h_ and *W*_exc_ are influenced by the cutting procedure, while the classical energy losses are unrelated to the cutting effect	[5,10]
*B*, total power losses	M400-50A, M800-65A, M270-35A/punching and laser	$\begin{array}{l} \left[\begin{array}{c} 1-\gamma_{1}\;\;\;\gamma_{1}\\ 1-\gamma_{2}\;\;\;\gamma_{2} \end{array}\right]\left[\begin{array}{c} B_{n}\\ B_{d} \end{array}\right]=\left[\begin{array}{c} B_{1}\\ B_{2} \end{array}\right],\\ p_{Fe}=c_{1}B+c_{2}B^{2} \end{array}$	- Pairs (*H*, *B*_n_) and (*H*, *B*_d_) were computed from experimental results obtained from two identical samples with different widths (*H*_1_, *B*_1_) and (*H*_2_, *B*_2_); - Values of *B*_1_, *B*_2_, *B*_n_, and *B*_d_ are determined for the same magnetic field strength value	[79,80]
*Μ*, *B*, *H*, *p*_Fe_	M235-35A/guillotine	$\begin{array}{l} J\left(H,x\right)=\mu_{0}\left(\mu_{r}\left(H,N=0\right)-\Delta \mu_{cut}\left(H\right)\eta \left(x\right)\right)H,\\ \eta \left(x\right)=1-\frac{x}{\delta }-a\frac{x}{\delta }\left(1-\frac{x}{\delta }\right),\\ p_{Fe}=a_{2}\left(x\right)J{\left(H,x\right)}^{2}f+a_{1}\left(1+a_{3}J{\left(H,x\right)}^{a_{4}}\right)J{\left(H,x\right)}^{2}f^{2}+a_{5}J{\left(H,x\right)}^{1.5}f^{1.5} \end{array}$	- Parameter δ is the degradation depth and is a function of *x*, which is considered as the distance from the cut edge; - The parameters Δμ_cut_ and η(*x*) are computed based on experimental measurements	[83]
μ, *p*_Fe_	M1000-65D/punching	$\begin{array}{l} \mu_{d}=\mu_{n}e^{\frac{-ax}{d}},\\ p_{Fe}=\frac{1-e^{-\eta \alpha }}{\eta \alpha }p_{d},p_{d}=aB^{\eta } \end{array}$	- The exponential variation in the magnetic permeability modeled the cutting effect, which consisted of μ decrease from the tooth center toward the cut edge	[81]
μ	NO20, M330-35A/punching	$\begin{array}{l} \mu_{d}\left(x,B\right)=\mu_{n}\left(B\right)+\left[1-\mu_{n}\left(B\right)\right]\left[2\delta \alpha \left(B\right)\left(1-e^{\frac{-1}{\delta \alpha \left(B\right)}}\right)-e^{\frac{-1}{\delta \alpha \left(B\right)}}\right],\\ \delta =\frac{e}{w} \end{array}$	- Parameter *e* represents sheet thickness, *w* is the sample width, while degradation parameter α(*B*) is experimentally determined; - The results of the model proved that the investigated NO FeSi are strongly dependent on the punching process, which was applied; - The model was considered a semi-empirical one, and the authors concluded that it could be useful in high-speed motor production	[91]
μ, *p*_Fe_	M400-50A/punching, laser, and EDM	$\begin{array}{l} \mu \left(H,x\right)=\mu_{n}\left(H\right)-\Delta \mu \left(H\right)e^{-ax},\\ p_{Fe}\left(B,x\right)=\left(K_{h0}+\Delta K_{h}\left(B\right)e^{-bx}\right)B^{\alpha \left(B\right)}f+K_{e}B^{2}f^{2}+\left(K_{ex}\left(B\right)e^{-cx}\right)B^{1.5}f^{1.5} \end{array}$	- Parameters Δμ(*H*) and *a* are experimentally computed	[92,93,94]
μ, *p*_Fe_	M400-50A/punching	$\begin{array}{l} \mu \left(H,x\right)=\mu_{n}\left(B\right)\left(1-e^{-\tau x}\right)+\mu_{d}\left(B\right)e^{-\tau x},\\ p_{Fe}\left(B,x\right)=\left(k_{h,n}\left(1-e^{-\tau_{h}x}\right)+k_{h,d}e^{-\tau_{h}x}\right)B^{2}f+\left(k_{e,n}\left(1-e^{-\tau_{e}x}\right)+k_{e,d}e^{-\tau_{e}x}\right)B^{2}f^{2} \end{array}$	- The exponential variation modeled the loss variation in the affected by cutting magnetic core; - The authors supposed that both hysteresis and total eddy current losses are influenced by the cutting procedure	[87,88]
*M*, magnetic susceptibility χ, *p*_Fe_	M270-50A/punching	$\begin{array}{l} M=\chi_{v}HM=M_{s}\left[\coth\left(\frac{H}{a}\right)-\frac{a}{H}\right],\\ a=\frac{M_{s}}{3\chi_{0}},\chi_{0}=\left(\chi_{n}-\chi_{d}\right)e^{-e^{-\left(\frac{x-d_{0}}{\beta_{0}}\right)}}+\chi_{d},\\ p=C_{hyst}fB+C_{exc}{\left(fB\right)}^{3/2}+\left(a_{ec}f^{3/2}+b_{ec}\right)\frac{\sigma {\left(\pi e\right)}^{2}}{6}{\left(fB\right)}^{2} \end{array}$	- Magnetization was modeled based on the Langevin function; - Saturation magnetization *M*_s_ is assumed that it remained unaffected by the cutting procedure; - Parameter χ_0_ represents the initial magnetic susceptibility influenced by the mechanical cutting; - Quantity *a* is a material parameter; - Quantities *d*_0_ and β_0_ are parameters of the cumulative Gumbel distribution used to compute the deteriorated magnetic susceptibility, which is a function of the distance from the cut edge *d*; - The loss coefficients depend on the distance to the cutting-edge	[89]
**Numerical models**				
**Analyzed physical quantities**	**NO FeSi**	**Numerical formulation**	**Remarks**	**Ref.**
Magnetic flux density *B* maps, for motor models: electromagnetic torque, iron losses	M330-50A/punching	- FEM model for one NO FeSi strip single tooth - FEM model for permanent-magnet synchronous motor (PMSM) and synchronous reluctance machine (SynRel) - -software: ONELAB [103]	- A good accordance between simulated and measured *B*(*H*) dependence was evidenced; - Local observation proves that in highly saturated regions, *B* values are strongly influenced by the cutting procedure; - For PMSM, a 25–30% increase in iron losses was reported for punched properties of NOFeSi; in the same conditions, a 40% increase for SynRel was obtained	[86]
*B* distributions, specific iron losses	M330-50A/punching, EDM	- FEM model for ring geometries - software: FEMM 4.2	- The numerical simulations with the analytical approach implementation proved a good computation convergence of the results compared with experimental measurements	[84]
*B* maps, iron losses, magnetomotive force	-	- FEM model for 4 poles synchronous generator and 2 poles capacitor motor - software: FEMM 4.2	- The analytical degradation profile was implemented in a layered configuration to characterize the damaged area; - Relative permeability μ_r_ was set on a specific layer having a decreasing trend towards the cut edge	[90]
*B* maps, iron loss	M400-50A, M800-65A, M270-35A/punching	- FEM model of a stator lamination stack - software: FEMM 4.2 coupled with MATLAB 2015	- The power loss density of the stator lamination stack was computed based on numerical simulations and from datasheet obtained from the NO FeSi producers. The inclusion of the analytical approach in the FEM simulations led to much more accurate results compared with the experimental ones because the electrical steel manufacturer used limitations for some corrective factors	[80]
*B* maps, core losses, thermal analysis	M400-50A/punching and laser	- FEM and thermal network models of a 37 kW three-phase cage induction motor - software: in-house FEM software based on MATLAB 2021	- The authors noticed that a significant increase in the magnetic flux density occurred in the case of the magnetic core, in which the magnetic permeability cutting-dependent model was included	[92,93,94]
*B* maps, core losses, efficiency	M270-50A/punching	- FEM model of a 130 kW PMSM - software: Not specified	- The material model was introduced in a semi-analytical model of a PMSM, whose design is optimized. It was noticed that the stator core losses increased by 1.75 due to the cutting procedure, a fact that is linked to an increase in temperature with 5 °C in stator windings	[89]
Electromagnetic torque	B35AV1900/shearing	- FEM model of a PMSM - software: INFOLYTICA MagNet v7	- The deterioration of the magnetic properties due to the cutting effect led to a decreased value of magnetic permeability and an increase in the core losses; - By incorporating the cutting technology effect in the FEM model led to a difference of about 8% in the output power in comparison with the motor model, in which this effect was not considered; - All the simulations were performed under the hypothesis that the material had an isotropic behavior even after cutting procedure	[95]
*B* maps, electromagnetic torque, harmonic analysis for magnetic flux density	M330-50A/punching	- FEM model of a double stator axial flux induction motor - software: COMSOL Multiphysics v. 5.6	- A five-layer structure of 0.2 mm approximation of the damaged area was assumed; - An analytical model of magnetic permeability degradation was included in FEM; - A decrease in the magnetic flux density in the air gap and a 1.3% decrease in the electromagnetic torque were reported when the cutting technology effect was considered	[96]
*B* maps, torque-speed maps, total power loss-speed maps	NO30-1600/punching	- FEM model of a V-shape magnets PMSM and a double-layered aluminum stator winding - software: Ansys Maxwell 2021	- An eight layer structure for the damaged area where was implemented a relative permeability degradation model; - An increase between 14% and 20% of hysteresis power losses and between 2% and 12% of total power losses was observed	[97]
torque-speed graphs, *L_q_* and *L_d_* computation, maximum current	M270-35A/laser	- FEM model of a SynRel - software: Not specified	- The degradation profile of the material was considered by using different values of relative permeability on specific areas on rotor and stator cores; - It was noticed that the manufacturing process had a degradation effect, especially on the stator part, because of higher magnetization current, while in the rotor core, it led to a performance improvement by increasing the inductance ratio	[98]
Cogging torque, efficiency, *B* maps	M330-50A/punching	- FEM model of V-shape magnets 8-pole PMSM - software: FEMM 4.2 and SyMSpace for the automatization of the problem solver [104]	- A layer-based approach was used, in which a magnetic permeability analytical model was implemented; - The author concluded that the manufacturing technology impact should be incorporated into future optimization studies to obtain novel and innovative motors	[99]
*B* maps, specific iron losses for ring samples,	M330-50A/EDM and punching	- FEM model for a ring sample - FEM model of a PMSM - software: FEMM 4.2	- The damaged material *B*(*H*) dependence was analytically implemented by the exponential degradation function of the magnetic permeability; - The tolerance analysis developed in the paper demonstrated that the consideration of sheet damage near the cut edge had an influence on the computation accuracy	[100]
Relative magnetic permeability maps, *B* maps in motor tooth, cogging torque	M330-50A/punching	- FEM model for one NO FeSi strip single tooth - FEM model for the 10-pole 12-tooth segmented stator of a PMSM - software: ONELAB [103]	- It was noticed that the damage to the teeth’ magnetic properties determined the existence of extra low-order harmonics in the motor cogging torque; - The authors concluded that the teeth position played an important role when punching parameters were included	[101]
*B* maps	-	- FEM model for 3 phase 37 kW cage induction motor - software: in-house FEM program	- The authors tested the application of mixer-order elements to include the cutting effects; - Second-order nodal triangular elements were applied in the vicinity of the cut edge, and transition/first-order elements were used for the remaining domain; - It was concluded after all the necessary simulations were performed that the mixed-order elements generated more accurate results in comparison with the classical finite-element approach	[102]

One may conclude that the numerical methods presented in Table 4 exhibit advantages and drawbacks. It can be noticed that many FEM approaches are based on multi-layered methods, in which the investigated strip is supposed to be cut using the same technology as intended for the magnetic core of the electrical motor. In addition, the power or energy losses and normal magnetization curves have to be experimentally determined in accordance with the existing standards on ring or strip samples, adjusted to the same width value as the motor geometric parts. To exactly model the motor geometry and electromagnetic behavior to be included in the FEM models, the obtained parameters for different widths must be introduced. It can be mentioned as a disadvantage the fact that it can be applied only in the case of a pre-existing database containing experimental measurements for the magnetic properties as a function of sample width and cutting technology. Another significant drawback is the high density of the discretization mesh required for an accurate computation of physical quantities. In addition, the fast change in electrical steel properties needs a low spatial resolution for the mesh, which generates a longer computation time and high modeling effort. The extraction of the local loss values and normal magnetization curves, which are used in the FEM models, can become a difficult task when the experimental measurements are performed with digital devices that are able to provide only the average values of the magnetic parameters across the strip width. In order to remove the eddy current effects, one should correct the local needle measurements.

Many of the analytical models require the determination of some parameters that are characteristic of the specific alloy type and the imposition of magnetizing conditions, which make the analytical expression efficiently applied in the case of a given electrical steel. The presented approaches provide different magnetic quantities that are influenced by the cutting procedure. When it is required to be associated with the FEM analysis, it permits the motor designers to choose a proper path.

## 5. Practical Implementation of Different Cutting Technologies in Electric Motor Manufacture

This section will briefly describe some practical implementations of electric motors that consider methods other than mechanical cutting technology to manufacture the rotor and stator magnetic core laminations.

Sano et al. [105] verified the validity of a practical modeling approach for NO FeSi degradation by manufacturing two 4-pole and 24-slot PMSM motors, whose magnetic cores were prepared by EDM and shearing, respectively. For both models, the authors used 35A360 electrical steel. They measured the back electromotive force (EMF) at 1800 rpm by connecting the two machines at one shaft, working alternatively as a motor and as a servo motor. The differences found between a FEM model with six layers, in which they included experimental normal magnetization curves, and the practical set-up proved that back EMF versus electrical angle had, in both cases, almost the same figure. In addition, shearing and EDM led to similar variations that proved the cutting had no important effect on the back EMF. The FEM simulation demonstrated that the magnetic flux density distribution was not uniform, and a much more pronounced effect of the cutting procedure was evidenced. Boubaker et al. [106] performed an experimental study regarding the iron loss measurements under real function conditions for a PMSM motor (frequencies higher than 1 kHz and an elevated power-to-weight ratio of about 4 kW/kg) dedicated to the aerospace industry. Based on laser and EDM technologies, the stator magnetic core was made of NO20 or M270-35A. The authors experimentally determined the iron losses for the no-load state and over a large frequency range until 1400 Hz. The obtained results were compared to those given by a numerical simulation performed in ANSYS Maxwell. The main conclusion of this paper was that supplementary losses occurred due to cutting procedures, real functioning conditions, and assembly steps. The best manufacturing process for these motors consisted of cutting the material through EDM, bonding varnish insulation, and stress relief thermal treatment. Leitner et al. [107] investigated the effect of manufacturing influence on the magnetic properties of three different magnetic cores of sub-fractional horsepower motors (SFHP) used for automotive fans. The stator lamination stacks were prepared as follows: punched M250-35A steel with interlocking (M250-35A PI), N010 (NO10 LB), and M250-35A (M250-35A LB) cut through laser technology with bonding. To measure the torque, a rheometer was used. The authors concluded that the punched M250-35A lamination stator was highly influenced by the manufacturing parameters, having the highest value of hysteresis torque (M250-35A PI: 0.11 (mN∙m), M250-35A LB: 0.075 (mN∙m), NO10 LB: 0.09 (mN∙m)), the lowest cogging torque (M250-35A PI: 0.93 (mN∙m), M250-35A LB: 1.18 (mN∙m), NO10 LB: 1.12 (mN∙m)), and up to 40% increase in iron losses. It was concluded that the best motor behavior was obtained for M250-35A steel cut through laser technology. In the case of the M250-35A PI stator, an 8.6% cogging torque peak-to-peak value decrease was reported, concomitantly with 37.5% larger average hysteresis losses compared to the other two solutions. For the NO10-LB stator, in comparison with M250-35A LB, an increase of 19.3% in hysteresis losses was measured, and it was noticed that the iron losses were lower only for speeds above 2300 rpm. The main contribution of this study consisted of providing an analysis of stator lamination design based on magnetic performance quantities for small motors. Bayraktar and Turgut [108] analyzed the cutting technology influence on a 5.5 kW 3-phase induction motor performance by preparing the stator laminations based on wire EDM, punching, laser, and abrasive WJ. The authors determined the motor efficiency and total losses by modifying the applied load between 25% and 125% at industrial frequency. Then, friction and windage losses and iron losses were investigated at 50 Hz. The maximum friction and windage losses were noticed for abrasive WJ (15.37 W), followed by laser (10.46 W), EDM (8.25 W), and punching (0.89 W). The iron losses behaved similarly, with the highest value obtained for WJ (0.712 kW), then laser (0.664 kW) and EDM and punching with about 0.56 kW. The total losses reported at 100% load had the maximum value of 1.492 kW (abrasive-WJ), then decreased values of 1.458 kW (laser), 1.277 kW (punching), and 1.236 kW (wire-EDM), respectively. It was shown that the best value of efficiency (85.61%) was obtained for EDM, while the lowest value was 83.1% for abrasive WJ. In the case of punching and laser technologies, values of about 85.16% and 83.47% were determined. For all cutting technologies, the motor efficiency reached the maximum level at 100% loading and successively decreased in the 115–125% critical load range. The main conclusion of this study was that residual stresses and plastic deformation influenced the motor efficiency in the case of punching and abrasive WJ methods and by thermal stresses induced by wire-EDM and laser. Credo et al. [98] studied the impact of manufacturing stresses on multiple-rib synchronous reluctance motor performance. It was proved that the manufacturing procedure performed based on laser cutting did not impact the motor efficiency by a high amount. An important influence was present inside the maximum torque area, where the working conditions are near the material saturation point. A possible performance improvement due to the manufacturing effects was attributed to the high reduction in the q-axis inductance since the ribs of the SynRel are thinner than those of other motor parts. The authors concluded that the manufacturing effect is very important and must always be taken into consideration when such motors are designed.

**Figure 9:**
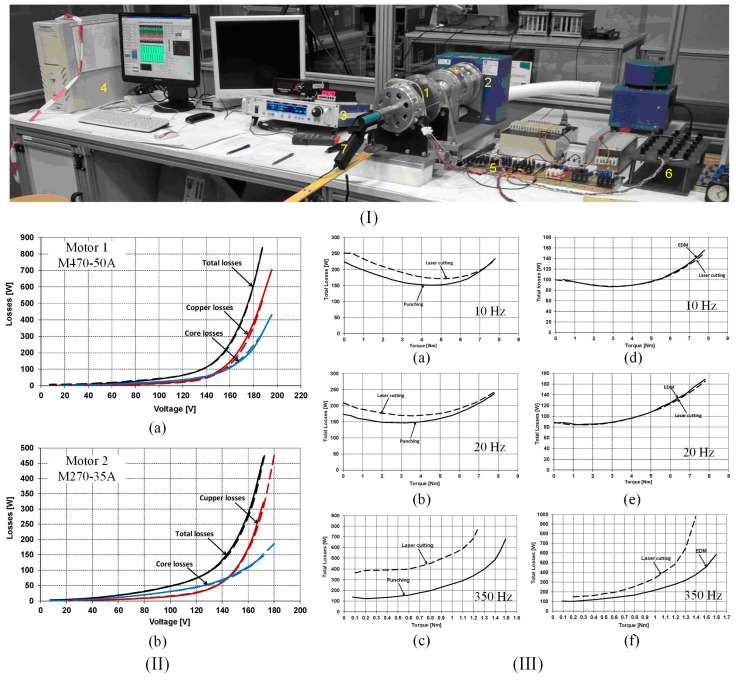
Experimental testing results of two types of motors having their magnetic cores cut through punching, laser, or EDM: (**I**) Experimental setup composed of 1—motor under test, 2—hysteresis dynamometer, 3—torque control system, 4—computer system, 5—current transducers, 6—resistive voltage divider, and 7—temperature monitoring system; (**II**) No-load analysis of total, windings, and core losses in the case of (**a**) Motor 1 with magnetic core manufactured from M470-50A steel grade (solid line—punching, dashed line—laser) and (**b**) Motor 2 manufactured from M270-35A electrical steel grade (solid line—EDM, dashed line—laser); (**III**) Total loss load characteristics for different cutting technologies and frequencies in the case of (**a**–**c**) Motor 1 and (**d**–**f**) Motor 2 designs [109]. Figures are licensed under CC—by 4.0.

Dems et al. [109] practically implemented two induction motor designs labeled Motor 1 and Motor 2, whose magnetic cores were made from M470-50A and M270-35A, respectively. For the first motor model, the laminations of the magnetic core were cut through punching (1P Motor) and laser (1L Motor), while in the case of the second model, the authors used EDM (2EDM motor) and laser (2L motor) technologies. All motor configurations were tested under no-load conditions at the industrial frequency of 50 Hz and load condition for the following values of the fundamental voltage harmonic frequency of 10, 20, and 350 Hz. The authors noticed that in the no-load condition case, the influence of the cutting method on stator current and different types of losses was minimal, with some increased losses for the laser cut configuration. In the case of load condition tests, it was observed that the laser technology determined a higher impact when compared to the mechanical in the case of Motor 1 (e.g., 10 Hz and load 4 Nm/total losses, Efficiency—laser: 176 W/38.8%, punching: 152 W/42.4%; 350 Hz and load 0.4 Nm/total losses, Efficiency—laser: 391 W/52.5%, punching: 133 W/76.5%), while for Motor 2, the differences between laser and EDM technologies were minimal (e.g., 10 Hz and load 4 Nm/total losses, efficiency—laser: 89 W/56%, EDM: 89 W/55%; 350 Hz and load 0.4 Nm/total losses, efficiency—laser 162 W/72%, EDM 118 W/78%) (Figure 9). Bayraktar and Turgut [23,110] investigated the effect of wire-EDM, abrasive WJ, mechanical punching, and laser technologies on 5.5 kW squirrel cage induction motor efficiency and total losses according to IEC 60034-2-1-1A [111]. The magnetic circuit was made from M400-50A electrical steel. From their studies, one can notice that the best efficiency and lowest loss values were obtained in the case of the wire-EDM motor, followed by the punching and laser cut cores within very closed limits. The worst motor behavior was found in the case of the abrasive WJ method.

Experimental measurements performed on electrical motors enable the producers to understand and identify the most adequate technologies related to high-efficiency motors and the modalities that must be applied to change some characteristics of cutting methods that are technologically and economically suitable for the motor series production. The studies summarized in this section provide the readers with an insight into how cutting technology influences the overall behavior of the motor by including all the material characteristics shown in the previous sections and give a step forward from the laboratory bench to technological transfer in the industry.

## 6. Conclusions

This paper represents an exhaustive review, which has as its main topic the influence of cutting procedures on non-oriented silicon iron steels’ macro- and microscopic features. The most common cutting methods used in the mass production of electric motors, such as mechanical and laser techniques, and the non-conventional technologies involved in the prototyping manufacture, such as abrasive water-jetting and electroerosion methods, are critically summarized. By analyzing the studies presented in Section 2 and Section 3 of the paper, one can conclude that the most important characteristics of the material, which determine the geometrical size of the damaged zone placed near the cut edge, are the elemental composition of the non-oriented silicon iron alloys and the strip thickness. In addition, in the case of mechanical cutting, the cutting speed, the cutting tool wear state; for laser, the type of laser; in the case of WJ, the nature of the abrasive particles and cutting speed; and for EDM, the type of the applied technology, i.e., based on wire or spark, should be considered as main factors with a strong influence on the damage of the material’s properties.

From Section 4, one can conclude that the magnetization processes, characteristic of the damaged zone width, are influenced by the value of the applied magnetic field strength. A link can be established between the damaged zone’s magnetic and mechanical properties because an increased hardness value and bad magnetic properties usually characterize the most affected areas by cutting. Regarding the crystallographic texture and magnetic domain structure extensively described in Section 3, one can also see a direct connection with the normal magnetization curve. When the crystallographic features of the electrical steels are modified near the cut edge, the movement of magnetic domains is highly influenced by pinning site formation and the magnetic domain shape deformation process. This fact is directly reflected in the normal magnetization curves, proving a more difficult magnetization process of the material associated with increased value of losses. In Section 4, there are summarized some analytical models developed by various research groups trying to explain the phenomenon of magnetic properties’ degradation near the cut edge by considering a mathematical approach relying on simple functions or statistical analysis. The width of the damaged zone can be estimated with the help of simplification hypotheses. Then, it can be included in different computations of the magnetic properties to model the material’s magnetic behavior in the damaged and undamaged cutting regions analytically. Some of these models were included in electromagnetic finite element analyses and proved to be a useful tool as a primary step in the electric motor design process, permitting the researchers to see and understand exactly the area where the cutting procedure influences the magnetic flux density as well as to estimate and compute the losses, cogging torque, or back electromotive forces in the case of different cutting procedures.

Section 5 presents examples of practical implementation of cutting process analysis in electric motor production. It can be seen that there are quite a limited number of papers that have included experimental studies and measurements on motors whose magnetic cores were cut through technologies other than classical punching or laser methods. By analyzing the existing literature, one recommends that the researchers investigate more how non-conventional technologies can be modified to be applied to the mass production of magnetic cores. For instance, it turned out that electroerosion led to high torques and reduced losses in electrical motors. Regarding the water-jet technology, some studies reported the worst magnetic behavior accompanied by the highest value of losses in real motors. Still, in the case of samples cut through this technology, a better magnetization process compared to laser and mechanical methods was noticed. In the near future, one foresees increased efforts by researchers to modify this technology to be applicable in small-series motor production because individual magnetic loss analysis demonstrated values comparable to those obtained through the electroerosion procedure. In this way, the thermal stresses due to EDM can be avoided. As future trends regarding the cutting technology for non-oriented electrical steel, the following ideas can be summarized: establishing a direct relationship between cutting variables and the chemical composition of alloys for a better understanding of the damaging effect, the study of deterioration mechanisms explained based on cutting stress influence on magnetic domain shapes, the correlation between surface roughness and the deterioration of magnetic properties in the damaged zone, finding a relationship between NO FeSi grain sizes and magnetic properties in the cut-edge vicinity, the development of simulations and modeling routes to underline the impact of process parameters to adjust the material microstructure to exhibit better mechanical and magnetic properties at the cut edge, and finding a direct link between the total losses and cut input parameters to adjust them to control the level of losses.

One important research gap that can be identified is the influence of the steel-cutting procedure on the cut-edge surface roughness. This parameter impacts the core welding process and the affected zone depth. Various cutting methods are expected to be linked to different roughness conditions, which affect the mechanical strength of the welding through the core axial direction. Discussing how different cutting technologies influence welding properties and surface roughness is important to adapt the cutting procedure in order to manufacture high-efficiency electric motors. Another gap is that all the analytical models strongly depend on geometry, material parameters, sample thickness, magnetic field strength, and processing properties. In the case of new materials characterized by different thicknesses, these analytical formulas must be adapted. In addition, attention should be devoted to an accurate FEM implementation of these equations for electric motor fabrication.

Overall, the information presented in this review paper can represent an insightful review for electric motor producers aiming to design and manufacture electric motors with improved efficiency to comply with the ever-increasing standards imposed by international norms and regulations. At the same time, one acknowledges that each aspect described in the present study must be individualized or corroborated with other emerging theoretical and practical advancements resulting from the constant quest for higher electric motor efficiency.

## Figures and Tables

**Table 1:** Advantages and drawbacks of electrical steel cutting technologies.

Cutting Technology	Advantages	Disadvantages
Mechanical (Guillotine)	Suitable for mass production; Can be used to cut multiple layers; Usually is accompanied by an automatic adjustment of the cut; Low-cost process	The cut-edge quality could decrease due to the bluntness of the guillotine; The cutting of corners can be difficult because of knife overcutting; The electrical steel sheet must be fixed; Cutting precise and small contours is difficult due to the fact that these are moved by the cutting force of the knife
Mechanical (Die cutting)	Low cost; Facile process; Less wasted material; Complex cut-edges shapes	Increased preparation time; The quality of the cut decreases during different production steps due to the bluntness of the blade; Difficult material positioning
Laser	Precise cutting method; High-speed process; Versatile technology; Exhibits no tool wear and has reduced running costs; Stable technology; It is characterized by a contactless process with no distortion and induced stresses on the workpiece; Exhibits a high design freedom; Clean process with a low accumulation of work rests; Can execute an extremely narrow cut; The cut-edges do not require dressing	The method could lead to cut-edge contour disturbances; The thermal stress of the steel with impact on mechanical and magnetic properties’ deterioration; Discoloration of the cut-edge; Supplementary thermal treatment necessity to relieve the induced stresses; Screening of beam; High costs for use and maintenance
Waterjet	Versatile technology; Complex shapes of the cut edges; Minimal distortion; Can be used in hazardous environments	The method usually needs the addition of abrasive particles; After the process, the electrical steel has to be dried; A filtering process of the water is necessary; The cut edges are rough; Induced thermal stresses; For cutting precision, some parts are placed in the water bath, and their fixation is necessary; Slow speed process; High capital costs; Safe handling of water jet; Noise; Abrasive cost cannot be recycled
Electrical discharge machining	Precise method; Burr-free cut edges; Cost-effective technology	Slow speed; Can cut only conductive materials; Must consider the effects of a charged environments

**Table 2:** Chemical composition of non-oriented electrical steels.

Alloy Grade	Chemical Element							Ref.
	Fe [%]	Si [%]	Mn [%]	Al [%]	P [%]	S [%]	C [%]	
M800-65A	96.4	1.43	0.58	0.215	0.049	0.005	0.033	[35]
M800-50A	98.3	1.177	0.213	0.129	0.043	0.005	0.0095	[35]
M400-65A	96.8	2.19	0.146	0.401	0.021	0.003	0.0095	[35]
M400-50A	97.1	1.99	0.183	0.376	0.021	0.003	0.0094	[35]
M350-50A	96.8	2.42	0.268	0.398	0.014	-	0.003	[39]
M330-35A	-	2.8	-	0.4	-	-	-	[41]
M300-35A	96.7	1.95	0.251	0.458	0.029	0.004	0.0094	[35]
NO20	96.5	2.31	0.185	0.341	0.033	0.004	0.0120	[35]
50WW800	-	1.80–2.20	0.15–0.35	0.20–0.4	≤0.030	≤0.006	≤0.0030	[42]
50WW470	-	0.25–0.70	0.30–0.70	0.25–0.50	0.050–0.110	≤0.008	≤0.0030	[42]
35WW300	95.92	3.1	0.26	0.65	0.005	0.002	0.01	[36,43]
35WW250	95.75	3.01	0.22	0.84	0.005	0.001	0.01	[36,43]
B35AV1900	96.1	3.13	0.29	0.44	0.11	0.001	0.01	[36,43]
NO FeSi	95.9	3.3	-	0.7	-	-	-	[37]
MS101	95.76	2.90	0.15	0.85	0.03	0.25	0.06	[38]
Fully processed NO FeSi with thickness of 0.35 mm	-	2.8	0.2	0.4	-	-	-	[40]

**Table 3:** Selected studies that investigated the residual stress apparition and distribution and the impact on material hardness.

Material	Cutting Technology	Residual Stress	Hardness	Ref.
M270-35A	Guillotine	Yield strength = 452 MPa, tensile strength = 590 MPa, fracture elongation 47.2%	-	[55]
FeSi with a thickness of 0.5 mm and 2.1 wt.%Si (Wuhan Iron and Steel Corp—WISCO, Wuhan, China) (SRA treatment applied after cutting procedure)	Shearing	The upper surface of the samples exhibited compression residual stresses (−300 MPa before SRA; −130 MPa after SRA), while the lower surface displayed tensile residual stresses (240 MPa before SRA, 85 MPa after SRA)	-	[56]
50WW470 cold-rolled non-oriented silicon steel from WISCO (SRA treatment applied for selected samples)	Punching	The residual stresses in NO FeSi are of compressive type. For the un-annealed and annealed samples, the maximum values of −340 MPa and −100 MPa, respectively, were found. The residual stress-affected zone was estimated at about 0.4–0.5 mm before the SRA	Testing conditions: Berkovich indenter, displacement 0.2 nm, load accuracy 3 nN, 23 measuring points at a distance of 25 μm. The maximum height located in the cross-section profile of the un-annealed sample was equal to −277.37 nm	[7]
NO FeSi with a hardness of HV150-220, thickness of 0.20–0.50 mm, and grain size of 70 μm	Shearing	Thicker strips showed more significant plastic strain and wider areas of increased hardness	The maximum hardness value near the cut edge was about 60% (HV0.5):154 and 50% (HV0.5):217. It gradually decreased to about 0% for a distance from a sheared edge of about 0.5 mm	[51]
M300-35, M470-50	Guillotine, laser	The guillotine-cut samples exhibited higher residual stresses (−179 MPa) in comparison with the laser-cut strips (−126 MPa)	For the guillotine-cut strip, the maximum values of hardness measured at the cut edge were equal to 350 HV_0.1_–M300-35 and 236 HV_0.1_–M470-50. The damaged zone width was about 200 μm. In the case of laser cut samples, the maximum values reached were 270 HV_0.1_–M300-35, and 160 HV_0.1_–M470-50. The damaged zone width was estimated at 70–80 μm	[57]
NO FeSi with 2.4 wt.% Si, thickness of 0.35 mm and 0.5 mm	Punching	In the area placed near the cut edge were numerically identified compression and tension stresses of about 250 MPa. Compression stresses in the cross-sectional area were determined in the middle of the blank. The shear stress had low values compared to compression and tension stresses	The maximum value of mean normalized hardness is about 1.7 in the case of a worn cutting tool at 0.15 m/s measured near the cut edge. The minimum value of 1.52 was noticed for a sharp cutting tool at 0.04 m/s	[6]

## Data Availability

Not applicable.
